# Broad Spectrum epidemiological contribution of cannabis and other substances to the teratological profile of northern New South Wales: geospatial and causal inference analysis

**DOI:** 10.1186/s40360-020-00450-1

**Published:** 2020-11-12

**Authors:** Albert Stuart Reece, Gary Kenneth Hulse

**Affiliations:** 1 Division of Psychiatry, University of Western Australia, Crawley, Western Australia 6009 Australia; 2 School of Medical and Health Sciences, Edith Cowan University, Joondalup, Western Australia 6027 Australia; 3 Brisbane, Australia

**Keywords:** Cannabis, Teratology, Cardiovascular defects, Atrial septal defect, Gastroschisis, Exomphalos, Genotoxicity

## Abstract

**Background:**

Whilst cannabis commercialization is occurring rapidly guided by highly individualistic public narratives, evidence that all congenital anomalies (CA) increase alongside cannabis use in Canada, a link with 21 CA’s in Hawaii, and rising CA’s in Colorado indicate that transgenerational effects can be significant and impact public health. It was therefore important to study Northern New South Wales (NNSW) where cannabis use is high.

**Methods:**

Design: Cohort. 2008–2015. Setting: NNSW and Queensland (QLD), Australia. Participants. Whole populations. Exposures. Tobacco, alcohol, cannabis. Source: National Drug Strategy Household Surveys 2010, 2013. Main Outcomes. CA Rates. NNSW-QLD comparisons. Geospatial and causal regression.

**Results:**

Cardiovascular, respiratory and gastrointestinal anomalies rose with falling tobacco and alcohol but rising cannabis use rates across Queensland. Maternal age NNSW-QLD was not different (2008–2015: 4265/22084 v. 96,473/490514 > 35 years/total, Chi.Sq. = 1.687, *P* = 0.194). A higher rate of NNSW cannabis-related than cannabis-unrelated defects occurred (prevalence ratio (PR) = 2.13, 95%C.I. 1.80–2.52, *P* = 3.24 × 10^− 19^). CA’s rose more potently with rising cannabis than with rising tobacco or alcohol use. Exomphalos and gastroschisis had the highest NNSW:QLD PR (6.29(2.94–13.48) and 5.85(3.54–9.67)) and attributable fraction in the exposed (84.11%(65.95–92.58%) and 82.91%(71.75–89.66%), *P* = 2.83 × 10^− 8^ and *P* = 5.62 × 10^− 15^). In multivariable geospatial models cannabis was significantly linked with cardiovascular (atrial septal defect, ventricular septal defect, tetralogy of Fallot, patent ductus arteriosus), genetic (chromosomal defects, Downs syndrome), gastrointestinal (small intestinal atresia), body wall (gastroschisis, diaphragmatic hernia) and other (hypospadias) (AVTPCDSGDH) CA’s. In linear modelling cannabis use was significantly linked with anal stenosis, congenital hydrocephalus and Turner syndrome (ACT) and was significantly linked in borderline significant models (model *P* < 0.1) with microtia, microphthalmia, and transposition of the great vessels. At robust and mixed effects inverse probability weighted multivariable regression cannabis was related to 18 defects. 16/17 E-Values in spatial models were > 1.25 ranging up to 5.2 × 10^13^ making uncontrolled confounding unlikely.

**Conclusions:**

These results suggest that population level CA’s react more strongly to small rises in cannabis use than tobacco or alcohol; cardiovascular, chromosomal, body wall and gastrointestinal CA’s rise significantly with small increases in cannabis use; that cannabis is a bivariate correlate of AVTPCDSGDH and ACT anomalies, is robust to adjustment for other substances; and is causal.

**Supplementary information:**

**Supplementary information** accompanies this paper at 10.1186/s40360-020-00450-1.

## Background

With major tobacco companies entering the cannabis market it is clear that cannabis commercialization is well under way [1]. Whilst much of the discussion relating to cannabis use and cannabis control is notably self-referential recent epidemiological reports suggest that intergenerational effects may be both significant and powerful enough to impact population-level health outcomes.

A recent report on Canada demonstrated that total congenital defects were three times more common in the northern Territories which smoked more cannabis than the Provinces and that the association was robust to socioeconomic adjustment [2]. A recent study from Colorado across the period of cannabis legalization showed that many defects rose parallel to increased cannabis consumption including all chromosomal defects (ACD), Downs syndrome and several cardiovascular defects including atrial septal defect (ASD) and patent ductus arteriosus (PDA), common defects which had not been previously linked with prenatal cannabis exposure (PCE) [3]. It was calculated that in Colorado over 11,000 extra defects occurred 2000–2014 related to increased cannabis use [3]. An Hawaiian study found that 21 defects were increased in mothers who were exposed only to cannabis [4].

Whilst some of these studies have used sophisticated geospatial modelling techniques [2] all epidemiological research is fundamentally associational in nature. However similar findings elsewhere strengthens the evidence base.

Northern New South Wales (NNSW, NSW) is a well known drug using and cannabis cultivation area 760 km from Sydney but only 180 km from tertiary pediatric care centres in Brisbane and 111 km from Southport both in Queensland (QLD). Although lying within New South Wales administratively many of its neonatal CA’s are evacuated to tertiary pediatric hospitals in Queensland under the Neonatal Retrieval Scheme (NRS) [5] and their data thus appears in Queensland statistics. This therefore presents an ideal opportunity to directly compare NNSW and Queensland neonatal epidemiology.

Our hypothesis was that cannabis use would be associated with increased congenital anomalies and was formulated prior to study commencement.

## Methods

### Data

Data on congenital anomaly rates for Queensland Health service areas including northern New South Wales was taken from the Congenital Anomaly Linked File (CALF) from Queensland Health [6]. Annual data by area has not been publicly released. Data on maternal age was from the QLD and NSW annual Mothers and Babies reports [7, 8]. CALF data includes numbers, rates and confidence intervals for the data. Drug use data for last month cigarette use, last month binge alcohol and last year cannabis use by area was obtained from the Australian Institute of Health and Welfare from the National Drug Strategy Household Survey (NDSHS) 2010 and 2013 [9] and averaged to obtain a mean rate by area across this period pursuant to our custom data request. Data was matched manually between drug use and congenital anomaly datasets. Areal shapefiles were taken from the Australian Government national website [10]. The northern coastal area of NSW was added on to the Queensland Health shapefile. This depiction of the NNSW catchment area is illustrative only and not intended to be exact as the geographic boundaries of the NRS are not defined [5].

Congenital anomalies were defined as cannabis related or not based on a literature review and recent reports [2, 3, 11–13] particularly [4].

### Sample characteristics

The samples were whole population samples and included all births in all health regions of Queensland. Hence inclusion and exclusion criteria were not applied as the samples were complete population samples. Sample capture in Northern New South Wales (NSW) appears to have been incomplete as some birth defects were not captured in the Queensland data and were managed in New South Wales. Hence the Northern New South Wales rates described in the present report clearly represents underestimates of the total rates. As the NSW and Queensland datasets are not directly comparable it is not possible to directly merge the two datasets to form a complete picture. This study limitation is discussed further in the limitations paragraph of the Discussion.

### Statistics

Data was processed in RStudio version 1.2.1335 based on R version 3.6.1 on 16th April 2020. Two-by-two tables were analyzed in package epiR using epi.2by2. Graphs were drawn with R-Base and ggplot2 [14] and in Microsoft Excel. Maps were drawn using the R packages ggplot2 and sf (“simple features”) [14, 15]. The software is freely available online and is directly loaded from inside RStudio software downloaded from the Comprehensive “R” Archive Network (CRAN). Licences for all R software are freely available with the R packages sourced from CRAN and its various mirrors internationally. Principal component analysis was conducted using the psych package. Linear regression was performed in Base-R. Batch extraction of all linear model coefficients by different defects was performed with broom and purrr packages. Correction of *P*-values for multiple testing was applied using the corrections of Holm, Bonferroni, Benjamini and Yekutieli, False Discovery Rate and Hommel as indicated. Links between neighbouring areas sharing an edge or corner (“queen”-relationships by analogy with chess moves) were derived with the poly2nb function from spdep and edited as indicated. This neighbourhood map was used to calculate the geospatial weights matrix for spatial regression.

Geospatial regression was performed with the spreml function from package splm [13, 16] using the derived spatial weights matrix. Spatial models may include the parameters phi, psi, rho and lambda for random effects in the error term, serial autocorrelation in the residuals, spatial errors, and autocorrelation in the spatial errors respectively. All spatial models used a full error structure of Kapoor Kelejian and Prucha [17] and had serially correlated remainder errors and random effects (sem2srre). The appropriateness of this error structure was formally tested by substituting various alternative forms and comparing results including the maximum likelihood ratio at model optimization (LogLik in the tables) and spatial Hausman tests and by examining the significance of the final model parameters [18]. Models were spatially lagged and not lagged as indicated.

Inverse probability weights (IPW) were derived with the ipw package in R using cannabis use as the exposure of interest, tobacco in the numerator and tobacco and alcohol in the denominator. IPW weights were then used in robust regression models conducted in the R package survey, and in mixed effects models in the R package nlme to generate datasets pseudo-randomized for cannabis exposure. This allowed causal relationships to be assessed. E-Values were calculated with the R package EValue to quantify the degree of association some unmeasured confounder would require with both dependent and independent variables to explain away the observed effect.

It was necessary to use various regression model structures for several reasons. Only spatial models can capture the spatial effects amongst the data, however they cannot be inverse probability weighted, so that causality can not be studied directly from them. Spatial models do provide a model variance which allows e-Values to be calculated from them. Robust marginal structural models can be inverse probability weighted, but do not provide model standard deviations so that e-Values cannot be calculated from them directly. Mixed effects models can be inverse probability weighted and also provide model standard deviations, but do not capture spatial effects and are also not robust in design. Hence for a full picture and understanding of the causal and spatial processes involved one needs to consider output from all of these various model structures together with their applicable e-Values.

For all regression models model reduction was by the classical method with sequential deletion of the least significant term. Missing data was casewise deleted at multivariable regression. *P* < 0.05 was considered significant.

### Data availability statement

Data including R software code have been made available online in the Mendeley Data Repository at 10.17632/cjzfyktz5m.1.

### Ethics

This study was approved by the Human Research Ethics Committee of the University of Western Australia on 15th April 2020 (No. RA/4/20/4724).

## Results

Input data is shown in an online supplementary csv file. Supplementary Table 1 provides comparative congenital anomaly data between QLD and NSW by both numbers and rates including defect relationship to cannabis [6]. Denominator data was calculated from the numbers and rates supplied in that file. It was verified from the annual QLD Health Mothers and Babies reports 2008–2015 which show 509,095 births in this period [8]. The “Interstate and Overseas” designation in the CALF file includes offshore islands such as Christmas, Norfolk, Cocos and Lord Howe Islands which together have a population of 4518. The prime catchment area of the NRS is Northern NSW which has a population of 296,531 [10]. Hence only 1.5% of the population in this designation is likely to come from outside NNSW. The view that the “Interstate and Overseas” designation refers primarily to NNSW is confirmed by QLD Health Ministerial correspondence (Minister Steven Miles, 05/04/2018). The denominator figure calculated for NNSW in this manner is 4800 births.

It should be noted that NNSW birth defect data also appears in NSW Health records [7]. One notes that the rates of congenital anomalies reported for this region in the NSW Mothers and Babies reports are about half those of the rest of the state. This is presumably related to the relocation of many cases into Queensland through the NRS. Queensland congenital anomaly rates are much higher than those reported elsewhere so it is not possible simply to combine NSW and QLD Health reports. Therefore this report is limited to consideration of the QLD Health CALF file only.

Drug use data is shown in Supplementary Table 2. It is noteworthy that the Richmond-Tweed NNSW area has a middle ranking for tobacco and alcohol use, but a first ranking for cannabis use.

Maternal age is a major factor bearing on congenital anomaly rates and it is known to be strongly linked with chromosomal anomaly rates. For years 2008–2015 4265/22084 (19.31%) births in NNSW were to mothers > 35 years compared to 96,493/490514 (19.67%; 18,581 missing maternal age data) in Queensland (Chi.Sq. = 1.687, df = 1, *P* = 0.194). This compares to 22,133/92242 (23.9%) of 2012 births in the rest of NSW (Chi.Sq. = 98.954, df = 1, *P* = 4.33 × 10^− 10^) indicating that NNSW mothers are younger in than elsewhere in NSW.

**Fig. 1:**
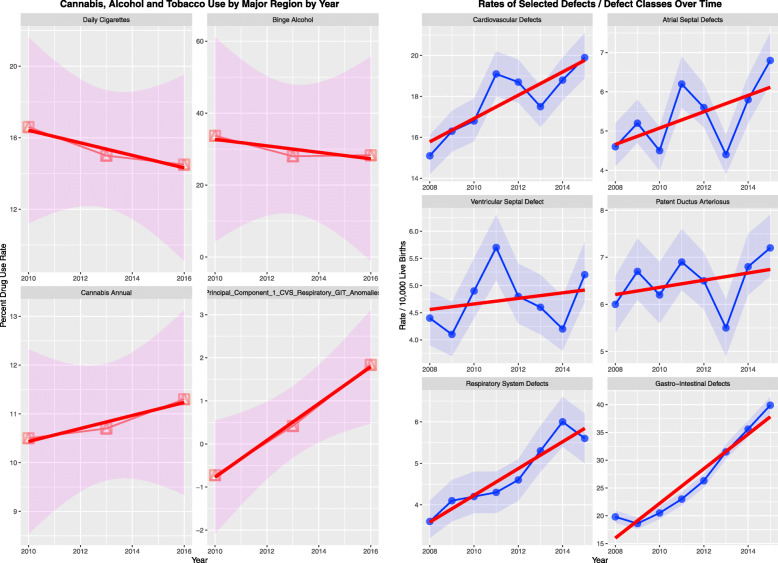
Trend analysis over time of (**a**) drugs and (**b**) various congenital defects and defect classes

Interestingly CALF Table 1 shows rises in the rates of several defects (Fig. 1) including CVS defects, atrial septal defect (ASD) and ventricular septal defect (VSD) which are highly significant (Supplementary Table 3). Intriguingly the mean incidence of daily smoking tobacco and high risk alcohol use dropped across this period and annual cannabis use rose from 10.5 to 11.3%. The principal component of the combination of cardiovascular, gastrointestinal and respiratory anomalies also rose significantly across this period. These data suggest that cannabis may be a more potent and more important teratogen than tobacco and alcohol.

**Table 1:** Results of geospatial additive regression by selected congenital anomalies - spatial error models

Parameters			Model		
Parameter	Estimate (C.I.)	*P*-Value	Parameters	Value	*P*-Value
***INTERACTIVE MODELS***					
spreml(Atrial_Septal_Defects ~ Tobacco * Binge_Alcohol * Cannabis)					
***Atrial_Septal_Defect***					
Binge_Alcohol: Cannabis	−0.58 (− 1.12, − 0.04)	0.0376	phi	0.0101	NA
Tobacco: Binge_Alcohol: Cannabis	0.03 (0, 0.06)	0.0417	psi	9.1E-08	1.0000
Tobacco: Cannabis	−0.09 (−0.18, 0)	0.0423	rho	−0.9960	0.0037
***CHROMOSOMAL_Defects***					
Tobacco: Binge_Alcohol: Cannabis	−0.01 (− 0.01, − 0.01)	0.0007	phi	0.0890	NA
Binge_Alcohol: Cannabis	0.05 (0.02, 0.08)	0.0021	psi	2.2E-05	0.9999
Tobacco: Cannabis	0.02 (0.01, 0.03)	0.0047	rho	−0.9805	0.0058
Tobacco: Binge_Alcohol	0.02 (0, 0.04)	0.0184			
***Diaphragmatic_Hernia***					
Cannabis	7.26 (3.21, 11.31)	0.0004	phi	0.0095	0.9964
Binge_Alcohol	7.33 (3.15, 11.51)	0.0006	psi	−1.7E-07	1.0000
Binge_Alcohol: Cannabis	−2.07 (−3.26, − 0.88)	0.0006	rho	0.9660	<2e-16
Tobacco: Cannabis	−0.22 (− 0.37, − 0.07)	0.0041			
Tobacco: Binge_Alcohol: Cannabis	0.06 (0.02, 0.1)	0.0051			
Tobacco	0.17 (0.04, 0.3)	0.0091			
***Downs Syndrome***					
Binge_Alcohol: Cannabis	0.03 (0.02, 0.04)	3.4E-05	phi	0.0571	NA
Binge_Alcohol	−2.33 (−3.7, − 0.96)	0.0008	psi	3.6E-05	0.9998
			rho	−0.6662	0.0626
***Gastroschisis***					
Cannabis	0.07 (0.03, 0.11)	0.0030	phi	0.0144	NA
			psi	1.8E-05	0.9999
			rho	−0.2503	0.5520
***Hypospadias***					
Tobacco: Cannabis	−0.13 (− 0.22, − 0.04)	0.0062	phi	0.0027	NA
Tobacco: Binge_Alcohol: Cannabis	0.04 (0.01, 0.07)	0.0084	psi	4.3E-05	0.9999
Tobacco: Binge_Alcohol	−0.35 (− 0.66, − 0.04)	0.0266	rho	−0.2080	0.6705
Tobacco	1.13 (0.11, 2.15)	0.0304			
***Patent_Ductus_Arteriosus***					
Cannabis	0.24 (0.01, 0.47)	0.0358	phi	0.0109	NA
Binge_Alcohol: Cannabis	−0.06 (−0.12, 0)	0.0526	psi	1.8E-06	1.0000
			rho	−0.9998	0.0022
***Small Intestinal Stenosis / Atresia***					
Cannabis	0.02 (0.01, 0.03)	0.0003	phi	0.0100	NA
			psi	1.3E-07	1.0000
			rho	−0.0089	0.9825
***Tetralogy_Fallot***					
Cannabis	0.09 (0.04, 0.14)	0.0007	phi	0.1102	0.9963
			psi	5.7E-05	0.9998
			rho	0.3074	0.3382
***Ventricular_Septal_Defect***					
Tobacco: Cannabis	−0.11 (− 0.21, − 0.01)	0.0328	phi	0.4947	NA
Tobacco: Binge_Alcohol: Cannabis	0.03 (0, 0.06)	0.0372	psi	1.2E-04	0.9997
Cannabis	2.93 (−0.23, 6.09)	0.0692	rho	−1.0000	0.0306
Binge_Alcohol: Cannabis	−0.85 (−1.79, 0.09)	0.0750

**Fig. 2:**
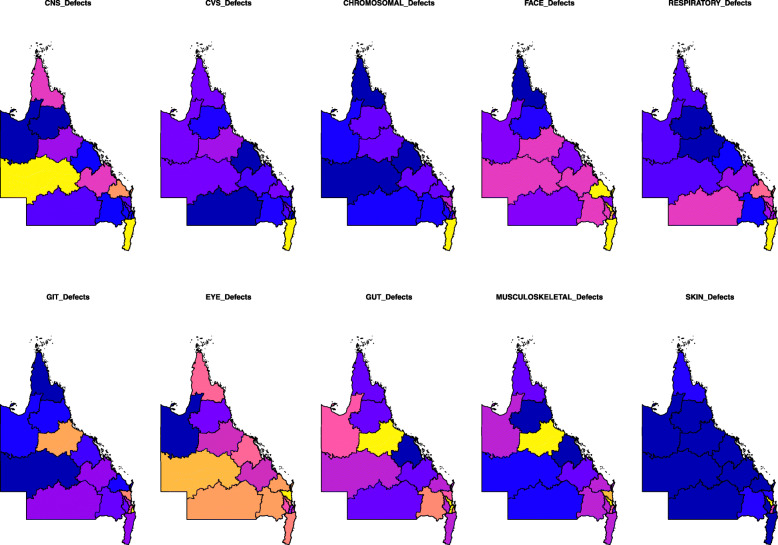
Choropleth maps of congenital anomaly class rates across QLD and NNSW. High rates are shown in yellow and low rates in dark blue. Maps were drawn using R package “sf” [15]

Figure 2 shows a qualitative choropleth map-graph for the major CA classes. The yellow zones reflect high incidence and dark blue low incidence.

**Fig. 3:**
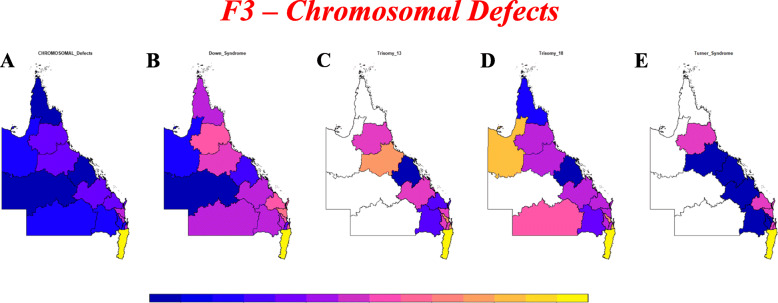
Choropleth maps of various chromosomal anomaly rates across QLD and NNSW. High rates are shown in yellow and low rates in dark blue. Maps were drawn using R package “sf” [15]

Supplementary Figs. 1–3 present choropleth maps of CA incidence by area. Figure 3 shows chromosomal anomaly incidence for which data is available.

**Fig. 4:**
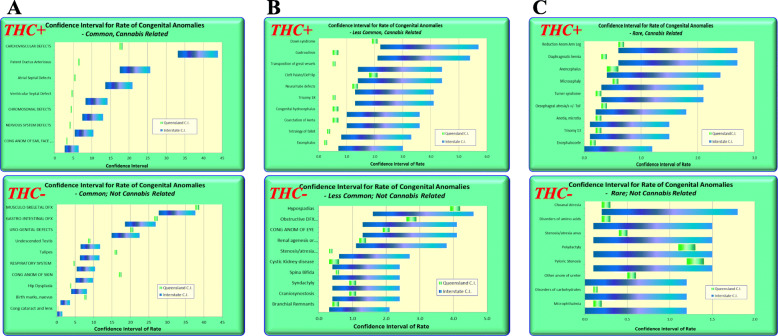
Confidence Intervals of cannabis-related and cannabis-unrelated congenital anomalies for (**a**) common, (**b**) intermediate frequency and (**c**) rare congenital anomalies

**Fig. 5:**
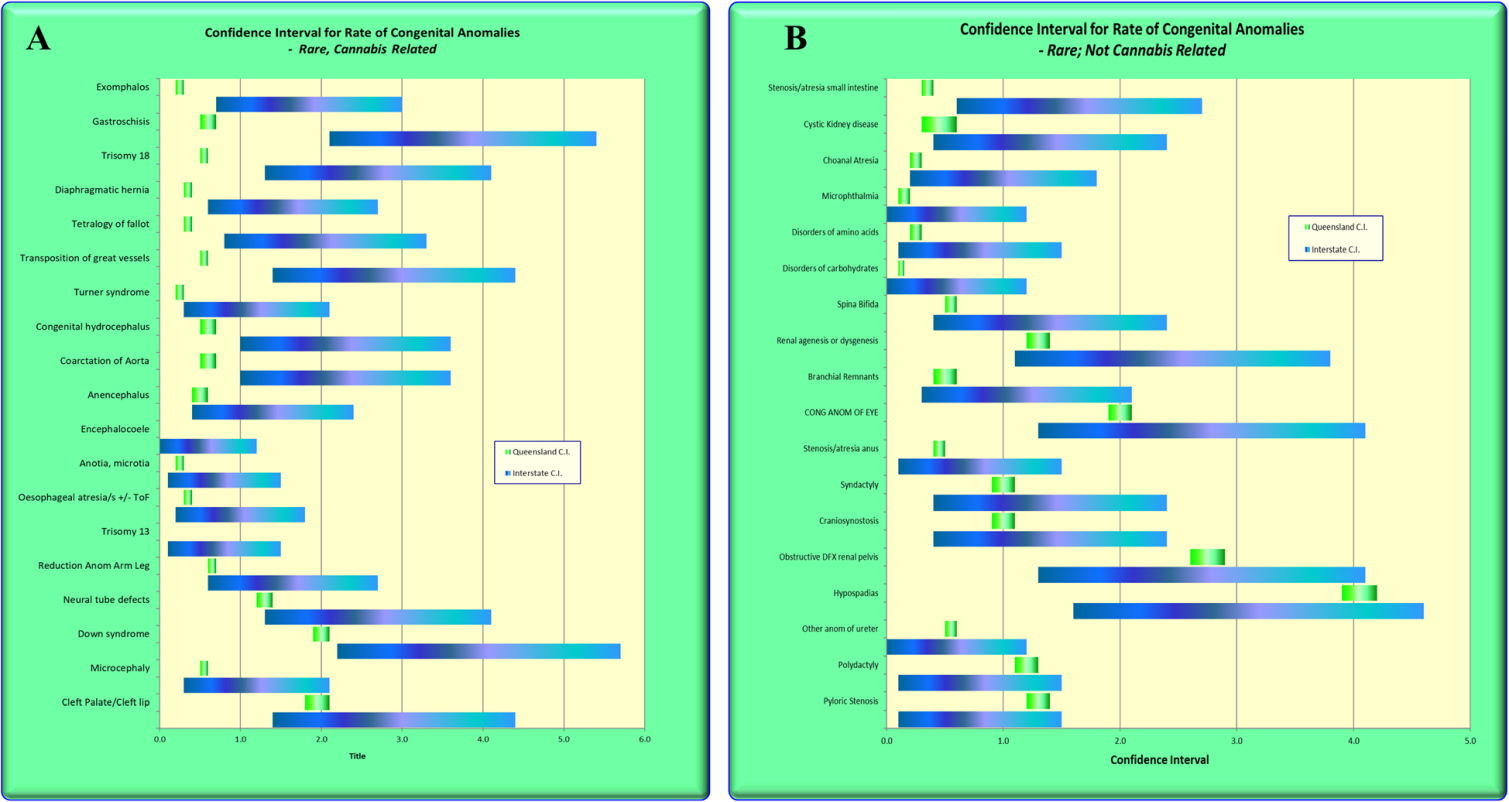
Confidence Intervals of rare cannabis-related and cannabis-unrelated congenital anomalies in more detail

Figure 4 was drawn in Excel and shows the confidence intervals from CALF for common, intermediate frequency and rare defects for cannabis-related (CRD) and cannabis not related (CNRD) defects. For most of the cannabis-unrelated defects the confidence intervals overlap. For most of the cannabis-related defects the confidence intervals either do not overlap, or are near the lower end of the QLD C.I.’s. Figure 5 expands this list for rare congenital anomalies and in general terms tends to continue this trend of non-overlapping confidence intervals or confidence intervals at the more extreme end of the range of the confidence intervals for cannabis-related defects.

**Fig. 6:**
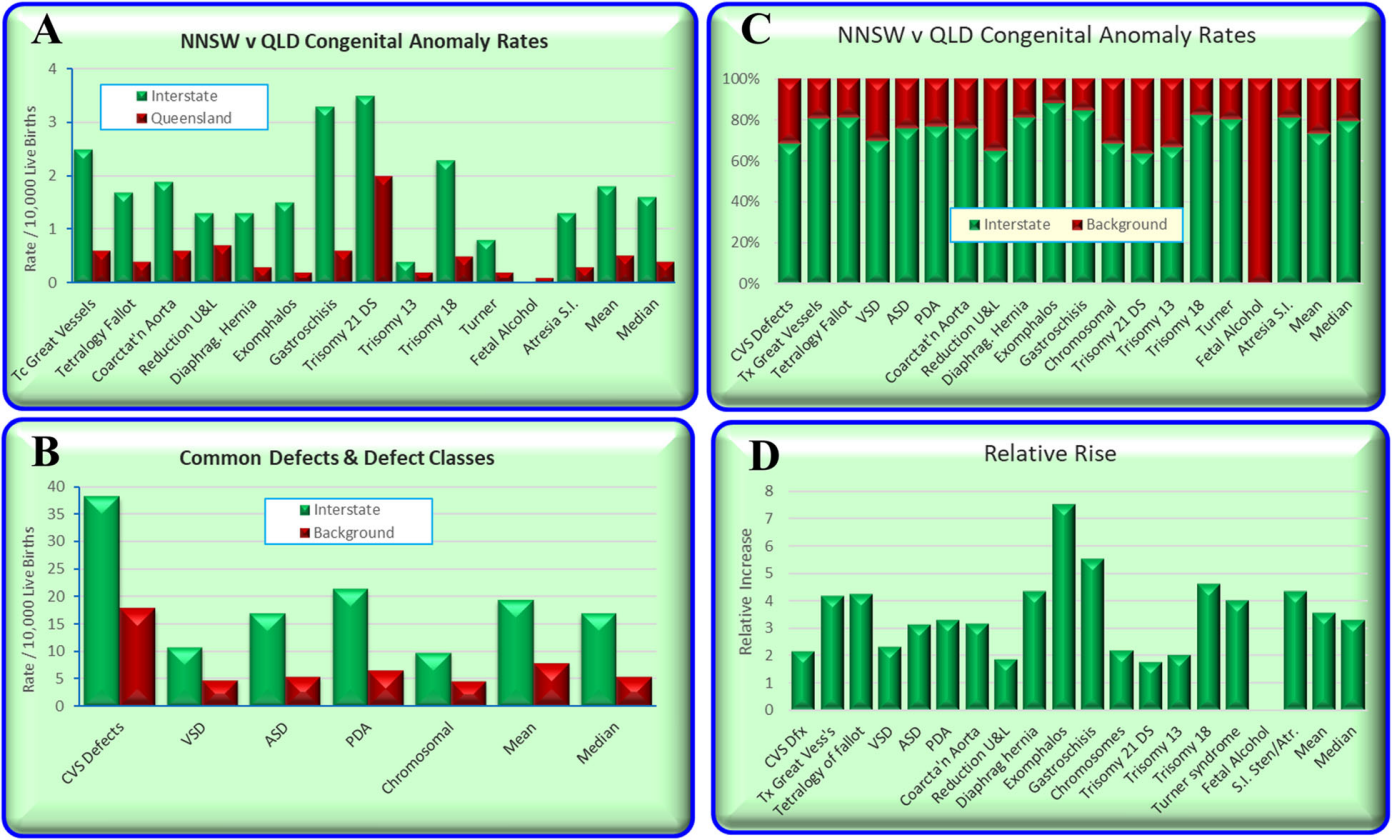
Relative Rates of Congenital Anomalies in Queensland v NNSW

Figure 6 compares the QLD and NNSW CA rates based on the relative rates quoted in the Queensland CALF file.

**Fig. 7:**
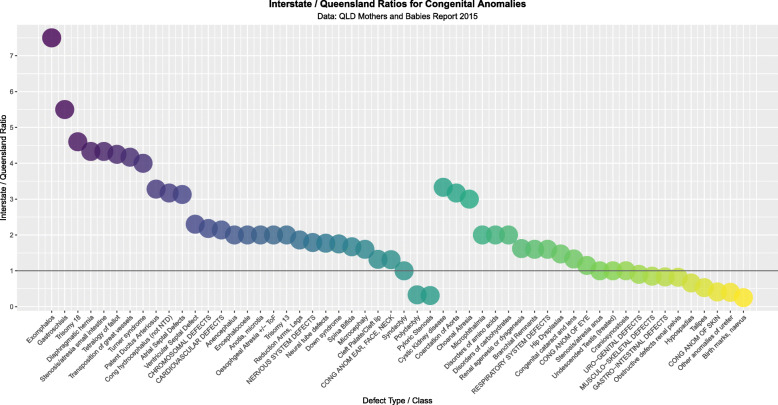
Relative Rate Ratios between Queensland and NNSW by relationship to Cannabis

**Fig. 8:**
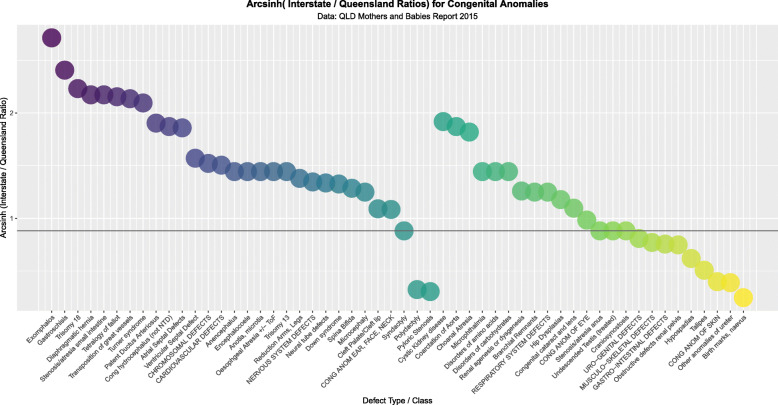
Log (Relative Rate Ratios) between Queensland and NNSW by relationship to Cannabis

Figure 7 compares all the rate ratios of defects using the quoted rates in the CALF file. Figure 8 makes a similar comparison with log rates and shows clearly that most of the cannabis-related defects are more common in NNSW.

CNRD were more common in QLD (23,737/509095, 4.66% v 185/4800, 3.85%, Chi Sq. = 7.002, df = 1, *P* = 0.0081). CRD were more common in NNSW (394/4800 8.21% v 16,346/509095, Chi Squ. = 376.86, df = 1, *P* = 6.01 × 10^− 84^). CRD were more common in NNSW than CNRD (394/4800 v 185/4800, Prevalence Ratio (PR) = 2.13 (95%C.I. 1.80–2.52), Chi Sq. = 80.284, *P* = 3.24 × 10^− 19^).

Supplementary Table 4 lists the PR’s, attributable fraction in the exposed (AFE) and attributable fraction in the population (AFP) along with their C.I.’s and applicable *P*-values for all defects and defect classes. They decline from exomphalos (PR = 6.29 (2.94–13.48), AFE = 84.11% (65.95–92.58%) and AFP = 4.71% (0.55–8.69%)) and gastroschisis (PR = 5.85 (3.54–9.67), AFE = 82.91% (71.75–89.66%) and AFP = 4.34% (1.79–6.82%)).

**Fig. 9:**
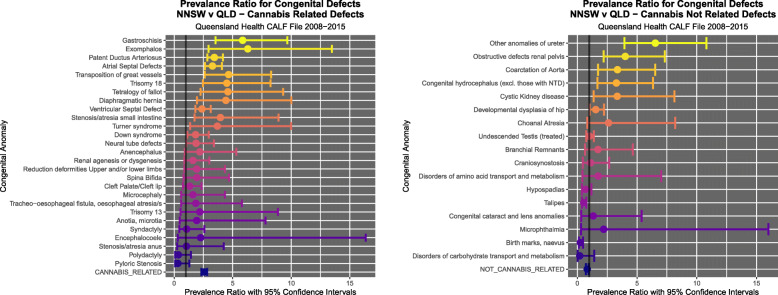
Prevalence Ratios (**a**) cannabis-related and (**b**) cannabis-unrelated congenital anomalies

**Fig. 10:**
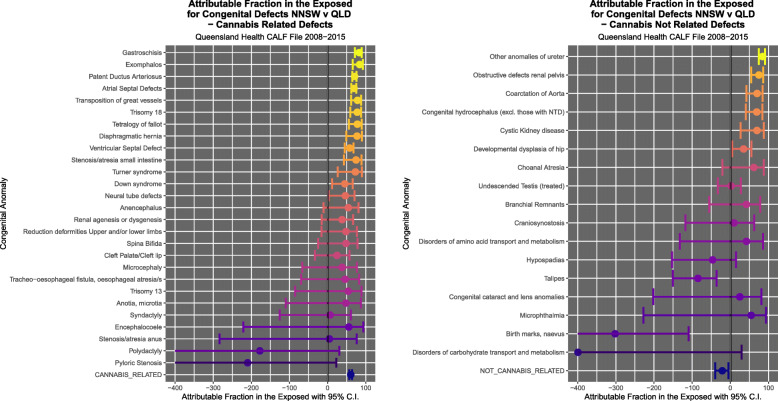
Attributable Fraction in the Exposed for (**a**) cannabis-related and (**b**) cannabis-unrelated congenital anomalies

**Fig. 11:**
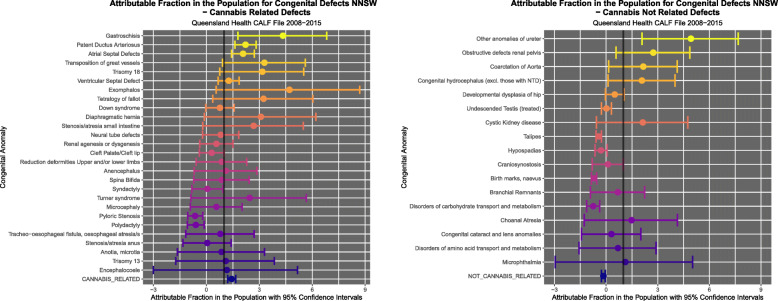
Attributable Fraction in the Population for (**a**) cannabis-related and (**b**) cannabis-unrelated congenital anomalies

**Fig. 12:**
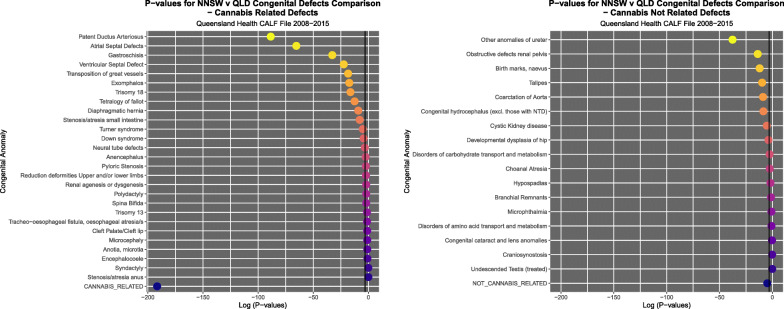
*P*-Values for (**a**) cannabis-related and (**b**) cannabis-unrelated congenital anomalies

Figure 9 illustrates the PR’s and C.I.’s for CRD and CNRD. Figure 10 shows the AFE’s and C.I.’s for CRD and CNRD. Figure 11 shows the AFP’s and C.I.’s for CRD and CNRD. *P*-values are illustrated in Fig. 12.

**Fig. 13:**
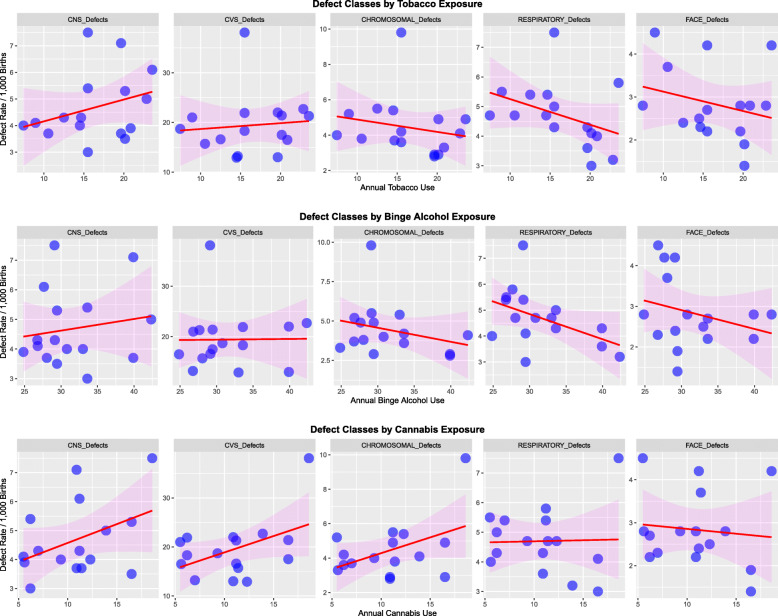
Response of five major congenital anomaly classes to (**a**) Tobacco, (**b**) Alcohol and (**c**) cannabis exposure

Figure 13 shows five main defect classes charted against the use of tobacco, binge alcohol and cannabis. Rising trends with cannabis seem to apply to CNS, cardiovascular and chromosomal anomalies.

**Fig. 14:**
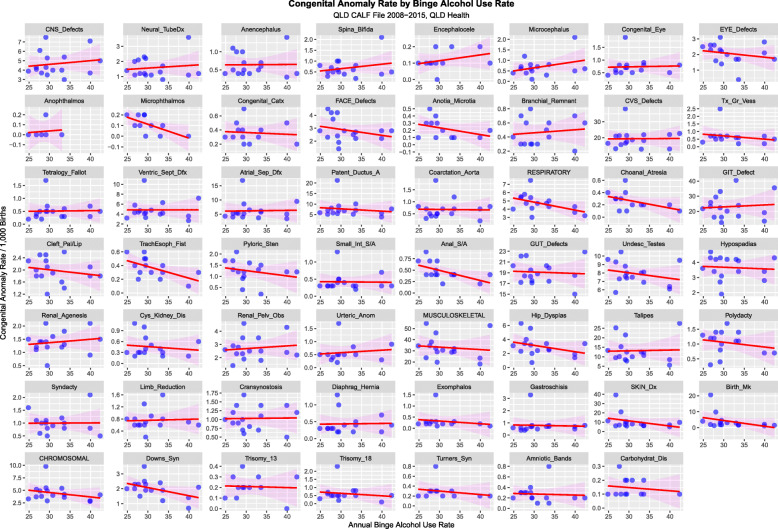
Congenital Anomaly Rate by Cannabis Use Rate – Ordered by Slopes of Least Squares Regression Lines

**Fig. 15:**
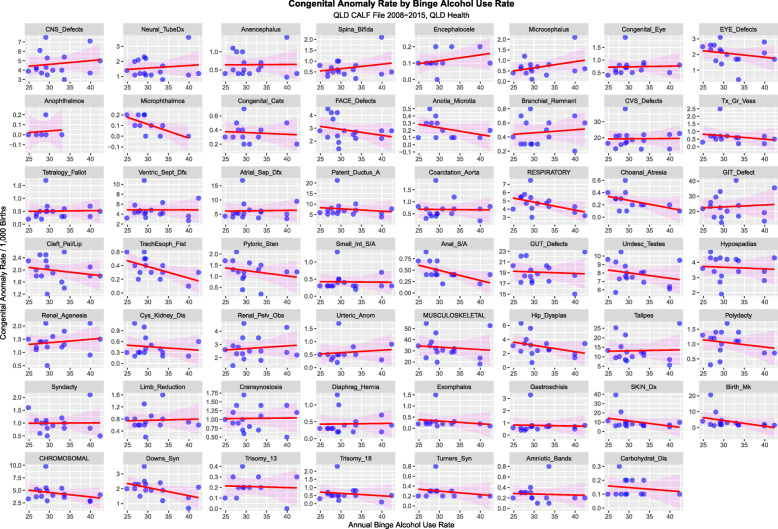
Congenital Anomaly Rate by Tobacco Use Rate – Ordered by Slopes of Least Squares Regression Lines

Figure 14 charts all 55 anomalies and anomaly classes against tobacco use. Figure 15 performs a similar function for binge alcohol.

**Fig. 16:**
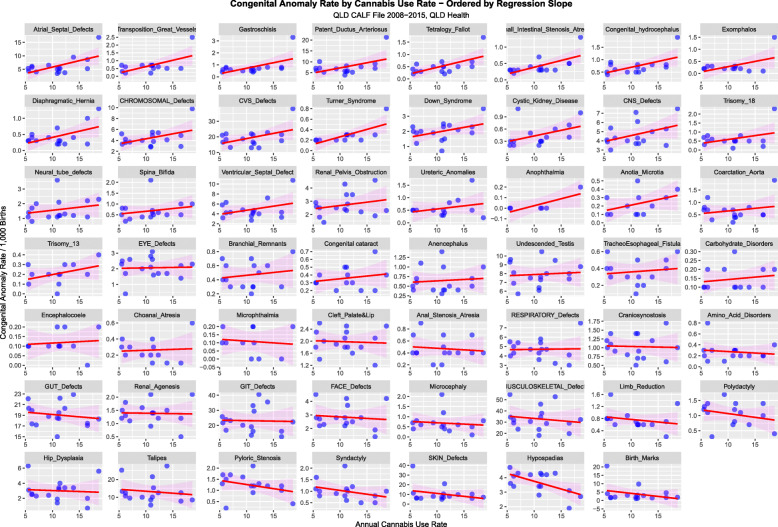
Congenital Anomaly Rate by Alcohol Use Rate – Ordered by Slopes of Least Squares Regression Lines

When a similar exercise is undertaken for cannabis exposure rising trends appear in several defects in the top three rows especially in cardiovasculature, chromosomal anomalies and body wall defects (Fig. 16).

Supplementary Table 5 lists the regression coefficients and their significance levels in ascending order of *P*-values for cannabis exposure. Supplementary Tables 6 and 7 show this table listed in order of ascending *P*-values for tobacco and alcohol respectively.

Supplementary Table 8 lists the significant output of a linear regression where the defect rate was related to additive terms of tobacco, binge alcohol use and cannabis use and includes all *P* < 0.3. This procedure selects 18 defects for further study. For biological and epidemiological reasons Trisomies 13 and 18 were also included.

Spatial analysis algorithms do not tolerate missing data. Hence linear regression was used to investigate 8 defects where spatial data was incomplete. Supplementary Table 8 shows the results of a model interactive in substances. Cannabis use was identified as being linked with several defects in this table including Turners syndrome.

**Fig. 17:**
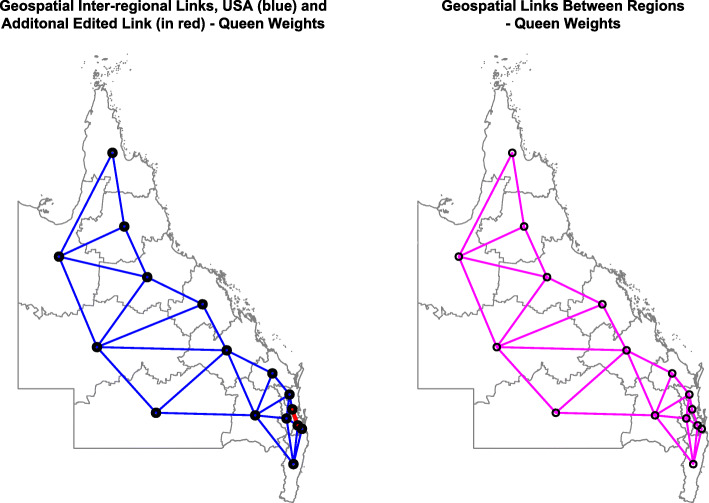
Geospatial Links Between regions – (**a**) Edited and (**b**) Final. Maps were drawn using R package “sf” [15]

Figure 17 shows the geospatial relationships which were derived from spdep::poly2nb and then edited to include all geospatial links.

Table 1 gives the results of geospatial regression for a model with additive terms in drug exposure. These are spatial error models and are not spatially lagged. In the additive model series cannabis is independently linked with all eight anomalies particularly cardiovascular (ASD, PDA and tetralogy of Fallot, ToF) and chromosomal (ACD and Downs syndrome), gastroschisis and small intestinal atresia.

Table 2 shows the results of an interactive spatial model and finds that cannabis is more strongly linked with these same defects. VSD is now positively associated as is diaphragmatic hernia which have both been previously noted to be cannabis-associated [19, 20].

**Table 2:** Results of geospatial interactive regression by selected congenital anomalies spatial error models

Parameters			Model		
Parameter	Estimate (C.I.)	*P*-Value	Parameters	Value	*P*-Value
***ADDITIVE MODELS***					
***spreml(log(Atrial_Septal_Defects) ~ DlyCigs11 + log(RiskAlc11) + Cannabis)***					
***Atrial Septal Defect***					
Cannabis	0.03 (0.00, 0.06)	0.0305	phi	1.01E-02	NA
			psi	−3.13E-06	0.9999
			rho	−7.64E-01	0.9400
***CHROMOSOMAL Defects***					
Cannabis	0.04 (0.01, 0.07)	0.0212	phi	1.05E-02	NA
			psi	2.99E-06	1
			rho	−4.66E-01	0.2291
***Downs Syndrome***					
Cannabis	0.09 (0.05, 0.13)	2.9E-05	phi	5.57E-02	NA
Binge_Alcohol	−2.02 (−3.35, − 0.69)	0.0029	psi	3.57E-05	0.9999
			rho	−6.85E-01	0.0883
***Gastroschisis***					
Cannabis	0.07 (0.03, 0.11)	0.0030	phi	1.44E-02	NA
			psi	1.78E-05	0.9999
			rho	−2.50E-01	0.552
***Hypospadias***					
Cannabis	−0.07 (−0.13, −0.01)	0.0166	phi	0.0419	0.9972
			psi	3.0E-05	0.9999
			rho	−0.6292	0.1224
***Patent Ductus Arteriosus***					
Cannabis	0.03 (0.01, 0.05)	0.0453	phi	1.01E-02	NA
			psi	−9.20E-06	1.0000
			rho	−1.0E+ 00	0.0025
***Small Intestinal Stenosis / Atresia***					
Cannabis	0.07 (0.03, 0.11)	0.0001	phi	1.09E-02	NA
			psi	6.74E-05	0.9998
			rho	−1.15E-02	0.9776
***Tetralogy Fallot***					
Cannabis	0.09 (0.04, 0.14)	0.0007	phi	1.10E-01	0.9963
			psi	5.67E-05	0.9998
			rho	3.07E-01	0.3382
***INTERACTIVE MODELS***					
spreml(Atrial_Septal_Defects ~ Tobacco * Binge_Alcohol * Cannabis)					
***Atrial_Septal_Defect***					
Binge_Alcohol: Cannabis	−0.58 (−1.12, − 0.04)	0.0376	phi	0.0101	NA
Tobacco: Binge_Alcohol: Cannabis	0.03 (0, 0.06)	0.0417	psi	9.1E-08	1.0000
Tobacco: Cannabis	−0.09 (− 0.18, 0)	0.0423	rho	−0.9960	0.0037
***CHROMOSOMAL_Defects***					
Tobacco: Binge_Alcohol: Cannabis	−0.01 (− 0.01, − 0.01)	0.0007	phi	0.0890	NA
Binge_Alcohol: Cannabis	0.05 (0.02, 0.08)	0.0021	psi	2.2E-05	0.9999
Tobacco: Cannabis	0.02 (0.01, 0.03)	0.0047	rho	−0.9805	0.0058
Tobacco: Binge_Alcohol	0.02 (0, 0.04)	0.0184			
***Diaphragmatic_Hernia***					
Cannabis	7.26 (3.21, 11.31)	0.0004	phi	0.0095	0.9964
Binge_Alcohol	7.33 (3.15, 11.51)	0.0006	psi	−1.7E-07	1.0000
Binge_Alcohol: Cannabis	−2.07 (−3.26, − 0.88)	0.0006	rho	0.9660	<2e-16
Tobacco: Cannabis	−0.22 (− 0.37, − 0.07)	0.0041			
Tobacco: Binge_Alcohol: Cannabis	0.06 (0.02, 0.1)	0.0051			
Tobacco	0.17 (0.04, 0.3)	0.0091			
***Downs Syndrome***					
Binge_Alcohol: Cannabis	0.03 (0.02, 0.04)	3.4E-05	phi	0.0571	NA
Binge_Alcohol	−2.33 (−3.7, −0.96)	0.0008	psi	3.6E-05	0.9998
			rho	−0.6662	0.0626
***Gastroschisis***					
Cannabis	0.07 (0.03, 0.11)	0.0030	phi	0.0144	NA
			psi	1.8E-05	0.9999
			rho	−0.2503	0.5520
***Hypospadias***					
Tobacco: Cannabis	−0.13 (− 0.22, − 0.04)	0.0062	phi	0.0027	NA
Tobacco: Binge_Alcohol: Cannabis	0.04 (0.01, 0.07)	0.0084	psi	4.3E-05	0.9999
Tobacco: Binge_Alcohol	−0.35 (− 0.66, − 0.04)	0.0266	rho	−0.2080	0.6705
Tobacco	1.13 (0.11, 2.15)	0.0304			
***Patent_Ductus_Arteriosus***					
Cannabis	0.24 (0.01, 0.47)	0.0358	phi	0.0109	NA
Binge_Alcohol: Cannabis	−0.06 (−0.12, 0)	0.0526	psi	1.8E-06	1.0000
			rho	−0.9998	0.0022
***Small Intestinal Stenosis / Atresia***					
Cannabis	0.02 (0.01, 0.03)	0.0003	phi	0.0100	NA
			psi	1.3E-07	1.0000
			rho	−0.0089	0.9825
***Tetralogy_Fallot***					
Cannabis	0.09 (0.04, 0.14)	0.0007	phi	0.1102	0.9963
			psi	5.7E-05	0.9998
			rho	0.3074	0.3382
***Ventricular_Septal_Defect***					
Tobacco: Cannabis	−0.11 (− 0.21, − 0.01)	0.0328	phi	0.4947	NA
Tobacco: Binge_Alcohol: Cannabis	0.03 (0, 0.06)	0.0372	psi	1.2E-04	0.9997
Cannabis	2.93 (− 0.23, 6.09)	0.0692	rho	−1.0000	0.0306
Binge_Alcohol: Cannabis	−0.85 (−1.79, 0.09)	0.0750			

Technical Abbreviations: *phi* Idiosyncratic component of the spatial error termpsi: individual time-invariant component of the spatial error termrho: spatial autoregressive parameter, lambda: spatial autocorrelation coefficient

A similar exercise is executed for spatially lagged spatial error (spatially autocorrelated with autocorrelated error components, SARAR) additive (Table 3) and interactive (Table 4) models with very similar results. In each case spatial error models were superior to combined SARAR models, as judged by the log maximum likelihood values, spatial Hausman tests and the largely non-significant lambda coefficients.

**Table 3:** Results of geospatial additive regression by selected congenital anomalies combined spatial error and spatial lag (SARAR) models

Parameter			Model		
Parameter	Estimate (C.I.)	*P*-Value	Parameters	Value	*P*-Value
***Atrial Septal Defect***					
Cannabis	0.04 (0.01, 0.07)	0.0078	phi	0.0706	NA
			psi	4.4E-05	0.9999
			rho	0.0373	0.9400
			lambda	−0.8526	0.0291
***CHROMOSOMAL Defects***					
Cannabis	0.04 (0.01, 0.07)	0.0227	phi	0.0072	0.9998
Tobacco	−0.03 (−0.06, 0)	0.0404	psi	2.1E-05	0.9999
			rho	0.2018	0.7175
			lambda	−0.2621	0.6308
***Diaphragmatic_Hernia***					
Cannabis	0.06 (0.01, 0.11)	0.0269	phi	0.0135	0.9994
			psi	1.4E-05	1.0000
			rho	0.6731	0.0333
			lambda	−0.7513	0.0436
***Downs Syndrome***					
Cannabis	0.09 (0.05, 0.14)	2.7E-05	phi	0.0135	NA
Binge_Alcohol	−2.02 (−3.34, −0.7)	0.0027	psi	−2.0E-05	0.9999
			rho	−0.7249	0.1285
			lambda	0.0633	0.8845
***Gastroschisis***					
Cannabis	0.07 (0.02, 0.11)	0.0029	phi	0.0116	0.9996
			psi	−2.6E-05	0.9999
			rho	−0.2004	0.7327
			lambda	−0.0568	0.9039
***Hypospadias***					
Cannabis	−0.09 (− 0.16, − 0.03)	0.0034	phi	0.2884	NA
			psi	−0.0001	0.9996
			rho	0.0213	0.9665
			lambda	−0.6716	0.0760
***Patent Ductus Arteriosus***					
Cannabis	0.03 (0, 0.06)	0.0365	phi	0.1043	0.9957
			psi	2.1E-05	0.9999
			rho	−0.5223	0.4700
			lambda	−0.76625	0.1705
***Tetralogy Fallot***					
Cannabis	0.08 (0.04, 0.13)	0.0005	phi	0.1269	NA
			psi	−0.0001	0.9998
			rho	0.5305	0.1006
			lambda	−0.3621	0.4032
***Ventricular Septal Defect***					
Tobacco	−0.03 (− 0.05, 0)	0.0166	phi	0.0068	0.9995
			psi	4.4E-06	1.0000
			rho	−0.9832	0.0135
			lambda	−0.3843	0.3813

**Table 4:** Results of geospatial interactive regression by selected congenital anomalies combined spatial error and spatial lag (SARAR) models

Parameter			Model		
Parameter	Estimate (C.I.)	*P*-Value	Parameters	Value	*P*-Value
***Atrial_Septal_Defect***					
Cannabis	0.04 (0.01, 0.07)	0.0078	phi	0.0706	NA
			psi	4.4E-05	0.9999
			rho	0.0373	0.9400
			lambda	−0.8526	0.0291
***CHROMOSOMAL_Defects***					
Tobacco: Cannabis	0.01 (0, 0.02)	0.0142	phi	0.0380	0.9971
Tobacco: Binge_Alcohol: Cannabis	0 (−0.01, 0)	0.0236	psi	2.1E-05	0.9999
			rho	−0.9999	0.0256
			lambda	0.5894	0.0866
***Diaphragmatic_Hernia***					
Cannabis	0.06 (0.01, 0.11)	0.0269	phi	0.0135	0.9994
			psi	1.4E-05	1.0000
			rho	0.6731	0.0333
			lambda	−0.7513	0.0436
***Downs Syndrome***					
Binge_Alcohol: Cannabis	0.03 (0.01, 0.04)	3.1E-05	phi	0.0158	NA
Binge_Alcohol	−2.33 (−3.69, − 0.97)	0.0008	psi	−2.8E-05	0.9999
			rho	−0.7121	0.1372
			lambda	0.0716	0.8688
***Gastroschisis***					
Cannabis	0.07 (0.02, 0.11)	0.0029	phi	0.0116	0.9996
			psi	−2.6E-05	0.9999
			rho	−0.2004	0.7327
			lambda	−0.0568	0.9039
***Hypospadias***					
Tobacco: Cannabis	−0.12 (− 0.2, − 0.04)	0.0035	phi	0.2455	NA
Tobacco: Binge_Alcohol: Cannabis	0.03 (0.01, 0.06)	0.0050	psi	−0.0002	0.9994
Tobacco: Binge_Alcohol	−0.33 (− 0.61, − 0.06)	0.0184	rho	0.0263	0.9544
Tobacco	1.09 (0.17, 2.01)	0.0201	lambda	−0.4710	0.1431
***Patent_Ductus_Arteriosus***					
Cannabis	0.26 (0.04, 0.49)	0.0208	phi	0.0100	0.9992
Binge_Alcohol: Cannabis	−0.07 (− 0.13, − 0.01)	0.0339	psi	1.7E-07	1.0000
			rho	−0.4196	0.5496
			lambda	−0.8473	0.1166
***Tetralogy_Fallot***					
Cannabis	0.08 (0.04, 0.13)	0.0005	phi	0.1269	NA
			psi	−5.2E-05	0.9998
			rho	0.5305	0.1006
			lambda	−0.3621	0.4032
***Ventricular_Septal_Defect***					
Cannabis	0.05 (0, 0.1)	0.0370	phi	0.0105	0.9993
Tobacco: Cannabis	0 (0, 0)	0.0420	psi	4.3E-06	1.0000
			rho	−0.9997	0.0360
			lambda	−0.2285	0.6320

One notes also that in a number of spatial error models spatial model parameters rho and lambda are noted to be highly significant. This therefore justifies the use of spatial models and also suggests that spatial factors are significant in considering clinical teratological patterns.

Table 5 summarizes the results of the above spatial models to facilitate comparisons between the various substances. The four spatial model structures are listed across the top of the Table and the various substances are listed in the rows. One notes that cannabis was independently predictive in 27 of the 36 models compared to only five each for tobacco and alcohol. Cannabis was involved in 19 interactive terms compared to ten and 13 for alcohol and tobacco respectively. It follows therefore that cannabis was involved in 46 terms compared to 15 and 18 for alcohol and tobacco respectively.

**Table 5:** Summary of geospatial regression model results by substance

Substance	Additive Error Models	Interactive Error Models	Additive Error-Lagged Models	Interactive Error-Lagged Models	Total Models
Number of Models	8	10	9	9	36
Cannabis Independently	8	5	8	6	27
Alcohol Independently	1	2	1	1	5
Tobacco Independently	0	2	2	1	5
Cannabis Interactively	0	12	0	7	19
Alcohol Interactively	0	6	0	4	10
Tobacco Interactively	0	8	0	5	13
Total Cannabis	8	17	8	13	46
Total Alcohol	1	8	1	5	15
Total Tobacco	0	10	2	6	18

Having demonstrated a strong associational relationship between drug exposure and several congenital anomalies the next issue of importance relates to the issue of whether the relationship was causal or not. Inverse probability weights were generated and used to derive a dataset pseudorandomized for cannabis exposure. Data was processed by robust interactive generalized linear modelling functions. As shown in Table 6 cannabis was significantly related to 18 anomalies either alone or in interaction with tobacco and alcohol.

**Table 6:** Robust generalized linear regression models

Parameter	Estimate (95%C.I.)	*P*-Value
***PC1***		
Cannabis	3.46 (0.58, 6.34)	0.0382
Cigarettes: Alcohol	0.45 (0.04, 0.85)	0.0521
Cigarettes	−1.52 (−2.89, − 0.16)	0.0510
Alcohol: Cannabis	−1 (−1.82, − 0.17)	0.0369
***PC1 as Arcsinh***		
Cannabis	2.89 (0.87, 4.92)	0.0172
Cigarettes: Alcohol	0.39 (0.11, 0.67)	0.0212
Cigarettes	−1.31 (−2.26, − 0.36)	0.0204
Alcohol: Cannabis	−0.85 (− 1.43, − 0.26)	0.0162
***Central Nervous System***		
Anencephalus		
Cigarettes: Cannabis	0.1 (0.04, 0.16)	0.0065
Cigarettes: Alcohol	0.55 (0.1, 1)	0.0384
Cigarettes	−1.81 (−3.32, − 0.31)	0.0399
Cigarettes: Alcohol: Cannabis	−0.03 (− 0.05, − 0.01)	0.0068
CNO		
Alcohol	2.8 (0.84, 4.76)	0.0189
Cannabis	2.6 (1.06, 4.14)	0.0078
Cigarettes: Alcohol: Cannabis	0.02 (0.01, 0.04)	0.0156
Cigarettes: Cannabis	−0.08 (− 0.14, − 0.02)	0.0194
Alcohol: Cannabis	− 0.79 (−1.25, − 0.34)	0.0067
***Cardiovascular System***		
ASD		
Cigarettes: Alcohol: Cannabis	0.12 (0.06, 0.18)	0.0051
Cannabis	8.4 (3.93, 12.88)	0.0062
Alcohol	21.3 (8.16, 34.43)	0.0130
Cigarettes	3.48 (1.07, 5.89)	0.0223
Cigarettes: Alcohol	−1.05 (− 1.78, −0.32)	0.0227
Alcohol: Cannabis	−2.5 (−3.8, −1.19)	0.0056
Cigarettes: Cannabis	−0.4 (− 0.61, − 0.19)	0.0055
VSD		
Cigarettes: Alcohol: Cannabis	0.04 (0.01, 0.07)	0.0242
Cannabis	3.38 (0.63, 6.13)	0.0366
Alcohol: Cannabis	−0.98 (−1.79, − 0.18)	0.0381
Cigarettes: Cannabis	−0.13 (− 0.23, − 0.03)	0.0239
PDA		
Cannabis	6.68 (1.28, 12.07)	0.0416
Cigarettes: Alcohol: Cannabis	0.09 (0.01, 0.16)	0.0504
Alcohol	16.71 (0.91, 32.5)	0.0719
Cigarettes: Cannabis	−0.29 (− 0.53, − 0.04)	0.0536
Alcohol: Cannabis	−1.98 (−3.55, − 0.4)	0.0392
Tetralogy Fallot		
Cannabis	0.08 (0.01, 0.15)	0.0410
***Gastrointestinal System***		
Cigarettes: Alcohol: Cannabis	0.02 (0.01, 0.03)	0.0031
Cigarettes	0.7 (0.27, 1.13)	0.0090
Cigarettes: Alcohol	−0.22 (−0.35, − 0.09)	0.0081
Cigarettes: Cannabis	−0.07 (− 0.11, − 0.04)	0.0029
Small Intestinal Stenosis or Atresia - Additive		
Cannabis	0.043 (0.004, 0.081)	0.0463
Small Intestinal Stenosis or Atresia - IR		
Cigarettes: Cannabis	0.002 (0, 0.005)	0.0692
***Genitourinary System***		
Cigarettes: Alcohol: Cannabis	0.01 (0, 0.01)	0.0113
Cigarettes	0.28 (0.08, 0.48)	0.0199
Cigarettes: Alcohol	−0.09 (− 0.15, − 0.02)	0.0195
Cigarettes: Cannabis	− 0.03 (− 0.04, − 0.01)	0.0103
Renal Pelvis Obstruction		
Alcohol: Cannabis	0.65 (0.1, 1.21)	0.0425
Cigarettes: Cannabis	0.08 (0, 0.15)	0.0656
Cigarettes: Alcohol: Cannabis	−0.02 (− 0.04, 0)	0.0614
Cannabis	−2.17 (−4.03, − 0.31)	0.0450
***Chromosomal Anomalies***		
Alcohol: Cannabis	0.02 (0, 0.05)	0.0678
Alcohol	−0.82 (−1.62, − 0.02)	0.0684
GIT		
Cigarettes: Cannabis	0.04 (0.01, 0.08)	0.0380
Cigarettes: Alcohol	0.34 (−0.01, 0.68)	0.0878
Cigarettes	−1.07 (−2.17, 0.02)	0.0862
Cigarettes: Alcohol: Cannabis	−0.01 (− 0.03, 0)	0.0357
Respiratory		
Cigarettes: Cannabis	0.01 (0, 0.02)	0.0927
Cigarettes: Alcohol: Cannabis	0 (−0.01, 0)	0.0497
Downs - Additive		
Cannabis	0.04 (0.01, 0.07)	0.0186
Alcohol	−1.29 (−2.43, − 0.15)	0.0464
Downs - Interactive		
Cigarettes	0.21 (0.01, 0.41)	0.0599
Cigarettes: Alcohol	−0.07 (− 0.13, − 0.01)	0.0441
Cigarettes: Alcohol: Cannabis	0.0007 (0.0002, 0.0013)	0.0198
***Body Wall***		
Musculoskeletal		
Cannabis	2.71 (0.93, 4.49)	0.0123
Cigarettes: Cannabis	−0.13 (− 0.22, − 0.05)	0.0113
Alcohol: Cannabis	− 0.8 (−1.32, − 0.27)	0.0130
Cigarettes: Alcohol: Cannabis	0.04 (0.01, 0.06)	0.0118
Gastroschisis		
Cigarettes	0.11 (0, 0.21)	0.0684
Cannabis	0.27 (0, 0.53)	0.0705
Cigarettes: Cannabis	−0.01 (−0.02, 0)	0.0920
Exomphalos		
Cigarettes: Cannabis	0.1 (0.04, 0.16)	0.0118
Cannabis	0.56 (0.14, 0.98)	0.0289
Cigarettes: Alcohol	1.03 (0.21, 1.85)	0.0357
Alcohol	−12.72 (−24.65, −0.8)	0.0661
Cigarettes	−3.2 (−5.78, − 0.61)	0.0386
Cigarettes: Alcohol: Cannabis	−0.04 (− 0.06, − 0.02)	0.0093
***Face***		
Cleft Palate / Lip		
Cannabis	0.57 (0.22, 0.92)	0.0092
Cigarettes: Alcohol	0.04 (0, 0.07)	0.0467
Alcohol: Cannabis	−0.12 (−0.2, − 0.04)	0.0128

This exercise was repeated with (non-robust) mixed effects modelling as such models in R have standard deviations associated with them, which is required in the E-values algorithm which follows. As shown in Table 7 similar results were obtained for 11 congenital anomalies.

**Table 7:** Mixed effects model results for selected congenital anomalies

Parameter	Estimate (95%C.I.)	*P*-Value
***Cardiovascular***		
**Atrial_Septal_Defect**		
Cannabis	0.053 (0.011, 0.096)	0.0280
**PDA**		
Cannabis	0.039 (− 0.004, 0.082)	0.0974
**Tetralogy_Fallot**		
Cannabis	0.079 (0.024, 0.133)	0.0135
***GIT***		
Cigarettes: Alcohol: Cannabis	0.022 (0.004, 0.041)	0.0362
Cigarettes	0.759 (0.064, 1.455)	0.0581
Cigarettes: Alcohol	−0.219 (−0.424, − 0.013)	0.0637
Cigarettes: Cannabis	−0.082 (− 0.145, − 0.019)	0.0285
**Small_Intestinal_Stenosis_Atresia**		
Alcohol: Cannabis	0.02 (0.008, 0.032)	0.0059
***GUT_Defects***		
Cigarettes	0.278 (0.043, 0.513)	0.0405
Cigarettes: Alcohol	−0.085 (−0.156, − 0.015)	0.0370
Cigarettes: Alcohol: Cannabis	0.008 (0.002, 0.014)	0.0295
Cigarettes: Cannabis	−0.027 (− 0.048, − 0.006)	0.0289
***Chromosomal***		
**Chromosomal**		
Cannabis	0.084 (0.019, 0.149)	0.0253
Cigarettes: Alcohol: Cannabis	−0.001 (− 0.002, 0)	0.0701
**Downs_Syndrome**		
Cigarettes	0.208 (0.024, 0.392)	0.0464
Cigarettes: Alcohol: Cannabis	0.001 (0, 0.001)	0.0636
Cigarettes: Alcohol	−0.071 (− 0.124, − 0.018)	0.0231
***Body Wall***		
**Musculoskeletal**		
Cannabis	2.686 (0.671, 4.701)	0.0241
Cigarettes: Alcohol: Cannabis	0.038 (0.01, 0.067)	0.0236
Alcohol: Cannabis	−0.79 (−1.385, − 0.195)	0.0246
Cigarettes: Cannabis	−0.131 (− 0.228, − 0.034)	0.0228
**Gastroschisis**		
Cannabis	0.065 (0.016, 0.115)	0.0207
**Exomphalos**		
Alcohol: Cannabis	0.042 (0.002, 0.082)	0.0596
Cigarettes: Cannabis	−0.006 (−0.011, 0)	0.0874

It is conceivable that the described relationships were related to some factor other than the measured covariates. E-Values quantitate the degree of association required of some unmeasured confounder with both cannabis exposure and the dependent variables to explain away the described effect. One notes that 1.25 is the value quoted in the literature as the threshold of interest in sensitivity analyses [21]. Table 8 lists 27 e-Values and finds that 21 minimum e-Values were greater than 1.25 and ranged up to 3.8 × 10^30^ for geospatial models and up to infinity for mixed effects models, making uncontrolled confounding unlikely.

**Table 8:** E-values for key regression parameters

Parameter	Table	Estimate (95%C.I.)	RR	E-Values
***Mixed Effects Models***				
lme(Congenital_Anomaly ~ Tobacco * Binge_Alcohol * Cannabis)				
**Cardiovascular Anomalies**				
Atrial_Septal_Defect				
Cannabis	Table 7	0.053 (0.011, 0.096)	1.15 (1.03, 1.29)	1.57, 1.20
**Tetralogy of Fallot**				
Cannabis	Table 7	0.079 (0.024, 0.133)	1.18 (1.05, 1.32)	1.63, 1.29
**Gastrointestinal Tract Anomalies**				
Cigarettes: Alcohol: Cannabis	Table 7	0.022 (0.004, 0.041)	1.3E+ 160 (3.5E+ 30, 5.1E+ 289)	Infinity, 6.9E+ 30
**Small_Intestinal_Stenosis_Atresia**				
Alcohol: Cannabis	Table 7	0.02 (0.008, 0.032)	1.05 (1.02, 1.09)	1.29, 1.16
**Genitourinary Tract Anomalies**				
Cigarettes: Alcohol: Cannabis	Table 7	0.008 (0.002, 0.014)	Infinity (6.9E+ 169, Infinity)	Infinity, Infinity
**Chromosomal Anomalies**				
Cannabis	Table 7	0.084 (0.019, 0.149)	1.32 (1.06, 1.64)	1.97, 1.33
**Downs_Syndrome**				
Cigarettes: Alcohol: Cannabis	Table 7	0.001 (0, 0.001)	1.002 (1.000, 1.004)	1.048, 1.009
**Body Wall Anomalies**				
**Musculoskeletal**				
Cannabis	Table 7	2.686 (0.671, 4.701)	Inf (Inf, Inf)	Infinity, Infinity
**Gastroschisis**				
Cannabis	Table 7	0.065 (0.016, 0.115)	1.16 (1.04, 1.30)	1.59, 1.24
**Exomphalos**				
Alcohol: Cannabis	Table 7	0.042 (0.002, 0.082)	1.07 (1.0045, 1.15)	1.36, 1.07
***Geospatial Models***				
spreml(Congenital_Anomaly ~ Tobacco * Binge_Alcohol * Cannabis)				
**Atrial_Septal_Defect**				
Binge_Alcohol: Cannabis	Table 1	−0.58 (−1.12,-0.03)	0.11 (0.13, 0.87)	17.99, 1.54
Tobacco: Binge_Alcohol: Cannabis	Table 1	0.03 (0,0.05)	1.11 (1.00, 1.22)	1.45, 1.07
Tobacco: Cannabis	Table 1	−0.09 (− 0.18,0)	0.71 (0.51, 0.99)	2.17, 1.12
***Tetralogy_Fallot***				
Cannabis	Table 1	0.09 (0.04,0.14)	1.21 (1.08, 1.37)	1.73, 1.39
**Ventricular_Septal_Defect**				
Tobacco: Cannabis	Table 1	−0.11 (− 0.21,− 0.01)	0.57 (0.34, 0.95)	2.90, 1.27
Tobacco: Binge_Alcohol: Cannabis	Table 1	0.03 (0,0.06)	1.17 (1.01, 1.37)	1.63, 1.11
**Patent_Ductus_Arteriosus**				
Cannabis	Table 1	0.24 (0.02,0.47)	2.63 (1.06, 6.51)	4.71, 1.34
**Chromosomal_Defects**				
Tobacco: Binge_Alcohol: Cannabis	Table 1	-0.01 (− 0.01,0)	0.97 (0.95, 0.99)	1.21, 1.13
Binge_Alcohol: Cannabis	Table 1	0.05 (0.02,0.09)	1.28 (1.09, 1.50)	1.88, 1.42
Tobacco: Cannabis	Table 1	0.02 (0,0.03)	1.07 (1.02, 1.12)	1.35, 1.16
**Downs Syndrome**				
Binge_Alcohol: Cannabis	Table 1	0.03 (0.01,0.04)	1.06 (1.03, 1.10)	1.33, 1.22
**Small Intestinal Stenosis / Atresia**				
Cannabis	Table 1	0.02 (0.01,0.03)	1.06 (1.03, 1.10)	1.31, 1.19
**Diaphragmatic_Hernia**				
Cannabis	Table 1	7.26 (3.21,11.31)	2.09E+ 09 (1.34E+ 04, 3.27E+ 14)	4.19E+ 09, 2.69E+ 04
Binge_Alcohol: Cannabis	Table 1	−2.07 (− 3.25,-0.88)	2.21E-03 (6.67E-05, 0.74)	9.00E+ 02, 26.56
Tobacco: Cannabis	Table 1	−0.22 (− 0.38,-0.07)	0.52 (0.33, 0.81)	3.28, 1.77
Tobacco: Binge_Alcohol: Cannabis	Table 1	0.06 (0.02,0.11)	1.20 (1.05, 1.36)	1.69, 1.30
**Gastroschisis**				
Cannabis	Table 1	0.07 (0.02,0.11)	1.17 (1.06, 1.31)	1.63, 1.30

## Discussion

This investigation presents many intriguing findings. Despite the several technical shortcomings of this dataset it is fascinating for the details and tantalizing clues which have been revealed. Importantly most of its major findings have been confirmed previously in other locations particularly in Colorado, Hawaii, Canada, and USA and by professional bodies such as AHA, AAP and CDC lending support to the strength of its principal results [2, 3, 11, 13, 19, 20].

NNSW has higher prevalence rates of the cannabis related anomalies: neural tube defects; small intestinal atresia; body wall defects: exomphalos, gastroschisis, diaphragmatic hernia; the cardiovascular disorders: ASD, VSD, PDA, tetralogy of Fallot, and transposition of the great vessels (TxGrVess); and the genetic disorders: all chromosomal disorders, Downs syndrome, Turners syndrome and trisomy 18. Amongst the defect classes cardiovascular, respiratory, and chromosomal anomalies were elevated. Some of these associations have been previously reported [3, 4, 22] and were seen in our unpublished analyses of US data.

QLD Health data showed that the NNSW CI’s for CRD’s were mostly non-overlapping or were at the extreme end of the QLD CI’s. CRD’s had higher rate ratios than CNRD’s.

Rising rates of cardiovascular, gastrointestinal and respiratory defects, and their first principal component were associated with falling rates of tobacco and alcohol use but rising cannabis use, just as was found in Colorado and USA [3].

At geospatial and linear regression the cardiovascular defects ASD, VSD, PDA, ToF, TxGrVess; the chromosomal defects ACD, Downs, Turners, Trisomy 13; the body wall defects gastroschisis, exomphalos, diaphragmatic hernia; the GI disorders small intestinal atresia and anal stenosis were all linked with cannabis exposure and for most cannabis exposure was an independent risk factor.

Rising rates of cannabis exposure were more strongly associated with cardiovascular, chromosomal, gastrointestinal and body wall defects than were rising rates of tobacco or alcohol exposure.

Analysis of this dataset by the formal techniques of causal inference analysis including inverse probability weighting and E-Values demonstrated that the described relationships fulfil the criteria for causal relationships.

These results show a striking concordance with epidemiological series from elsewhere. ASD, VSD, ToF, obstructive urinary disorders, hydrocephalus, anal anomalies and Downs syndrome were linked with PCE in a large Hawaiian series [4]. VSD has previously been linked with PCE [19]. Neural tube defects were noted to be elevated in a cannabis-related manner in Canada and Hawaii [4, 11]. ASD, PDA ACD and Downs were seen to rise in close temporal association with increased cannabis use in Colorado [3]. Exomphalos was implicated in animals [23, 24] and in some clinical series including in Queensland [25]. Transposition of the great vessels has previously been linked with *paternal* PCE [26]. Indeed in Canada total CA’s were linked with increased cannabis use after controlling for income and sociodemographic variables [2].

Many series implicate PCE in gastroschisis aetiology with a meta-analyzed bivariate O.R. = 4.12 (95%C.I. 3.45–4.91) [4, 27–32]. Our findings PR = 5.85 (3.54–9.67) contradict those of a 2011 NSW Health report on gastroschisis in this region [33] which erroneously applied an inflated Bonferroni correction to obviate a significant result. Indeed if the 9 cases reported in NSW [33] are added to the 16 cases reported in QLD the PR rises further to 9.13 (6.07–13.72).

Increasing reports from diverse sources indicate that the evidence is building that cannabis has significant teratological activities in humans in agreement with animal studies where many severe defects including oedema, exomphalos, phocomelia, spina bifida, myelocoele, exencephaly and foetal loss were documented [23, 24]. Concordant reports from Hawaii, Colorado and Canada suggest that the findings reported herein are indeed valid and are generalizable elsewhere. Given that likely half the NNSW congenital anomalies are reported internally within NSW [7] this suggests that the teratological situation in NNSW is indeed serious. Moreover some of the CA described here, especially chromosomal defects, are heavily therapeutically aborted antenatally again suggesting that the situation may well be much worse than our description suggests. Our analysis strongly implicates cannabis use as a likely underlying factor.

When one also considers the known epigenetic actions of cannabis [2, 12, 34–37] and its associations with developmental neurological dysfunction and autism [38–42] concerns relating to the intergenerational actions of cannabis are heightened.

From both the present data and from similar international analyses a number of important clinical implications arise. Notwithstanding its popular relatively benign image such analyses indicated not only that the potential teratological impacts of cannabis are significant but that they are likely causal in nature. Patients considering commencing a family should be encouraged to desist from all drugs prior to conception including cannabis. Patients who do fall pregnant and who are consuming cannabis should be encouraged to reduce and cease. Patients wishing to access treatment to assist with such withdrawal should be provided every encouragement and assistance to do so. Patients should be warned that the evidence base for the use of cannabis for most of its touted clinical indications is weak. Patients should be advised to avoid cannabis for morning sickness of pregnancy. Heavy cannabis smokers should be warned that cannabis hyperemesis can mimic hyperemesis gravidarum.

Moreover since the debate relating to cannabis is typically highly individualistic it seems prudent that medical professional organizations should partner with public health agencies and community groups to enlarge the focus of popular debate from the simply self-referential to a broader multigenerational perspective.

One major toxicological conclusion which follows directly from these studies is that access to cannabis should be highly restricted. Indeed such work calls into question the whole issue of the long term advisability of cannabis medicalization / legalization and the sustainability of such paradigms from a teratological perspective.

The present work has not considered neurological sequalae in the newborn and childhood as has previously been reported to overlap the autistic spectrum disorder and ADHD and thereby potentially play a major role in the modern widespread epidemic of these disorders [38–40, 43]. When such data is factored into consideration the imperatives for reconsideration and re-evaluation of cannabis legalization overall are largely increased.

### Comparison with alcohol

It is of interest to summarize and compare some of these results directly between cannabis and alcohol as the latter is a known human foetal teratogen and many learned bodies recommend strongly against tobacco exposure in pregnancy [44].

Figure 13 is a scatterplot of the frequency of the defect classes compared to the various substances. This Figure shows clearly that increasing cannabis use is associated much more strongly with several classes of congenital anomalies in this dataset than either tobacco or alcohol. The regression lines in this figure slope upwards much more strongly for cannabis for CNS defects, cardiovascular defects and chromosomal defects than for either of the other two substances.

Figures 14, 15 and 16 perform a similar role for each individual defect by the three substances tobacco, alcohol and cannabis. On the tobacco and alcohol scatterplots most of the regression lines are flat or falling. In contradistinction on the cannabis scatterplot many of the first 32 defects appear to be rising with positive slope. This is quantified in Supplementary Tables 5–7 for cannabis, tobacco and alcohol respectively. The slopes of the first 8 CA’s are significant and seven slopes are positive for cannabis. This compared to tobacco and alcohol where only two and four slopes are significant respectively and all the slopes are negative.

Table 1 presents the remarkable result that of eight additive spatial models cannabis is independently predictive for all eight defects and indeed tobacco and alcohol do not appear in final models. Similarly in Table 3 cannabis is independently predictive for eight of nine defects in additive SARAR spatial models. Alcohol only features in the model for Downs syndrome and its regression coefficient is negative. These differences are compared directly in Table 5, where as noted cannabis is implicated in 46 terms compared to 15 for alcohol and 18 for tobacco. Cannabis is implicated independently in 27 terms compared to five each for tobacco and alcohol.

The overall conclusion then from this detailed comparison must be that cannabis is a relatively more powerful or more potent human teratogen than alcohol.

### Causal inference

A classical criticism of correlative studies is that “correlation does not equal causation.” Judea Pearl, one of the leading causal statisticians in the world, has described this criticism as arising from what has been historically the “causalophobic” science of statistics [45, 46]. In relation to the present study the following points should be mentioned. Firstly to observe that an exposure and an outcome are associated not only statistically but also across space carries more weight than a simple statistical association. Secondly inverse probability weighting has been used in mixed effects and robust structural marginal models with very highly significant results. Inverse probability weighting is well established in the literature as transforming an observational study into a pseudo-randomized population from which casual inferences can properly be drawn. Thirdly we have used e-Values to quantify unmeasured confounding as a notorious source of extraneous confounding not controlled by the small number of covariates employed in the present analysis. E-Values provide a quantitative estimate of the degree of association required of any extraneous factor with *both* the exposure and the outcome to explain away the observed effect. Whilst in the literature e-Values above 1.25 have been stated to be noteworthy [21] our minimum e-Values ranged up to infinity in mixed effects models, and up to 5.2 × 10^− 13^ in spatial models. This finding implies both the causal nature of the relationship, and also that the inclusion of further parameters in the model would not obviate the described effects.

Hence our study demonstrates a causal relationship of drug and particularly cannabis exposure to several congenital anomalies. The causal relationship in this case is greatly strengthened by the existence of similar results from other places in the world as described [2–4, 11, 19] and the existence of a plethora of biological and epigenetic processes to account for these effects as mentioned [3, 11–13, 34, 35, 37, 39, 47–50].

It is further noted that the present findings fulfill all of the qualitative and quantitative Hill criteria for causality [51].

Our study has several strengths and limitations. Its strengths include access to whole population data for Queensland and a significant portion of the NNSW data. The CA rates and confidence intervals were already provided by QLD Health. The NDSHS is a nationally representative survey conducted every three years and the authoritative source for most Australian drug use data. Our analytical strategy combined CA with drug exposure data which is unusual and useful. We have employed a variety of powerful statistical techniques in this investigation including geospatial analysis, inverse probability weighting, mixed models and E-Values. Study limitations relate mainly to the remote location of the NNSW area close to the Queensland border and the small numbers of some anomalies reported. Losses due to treatment within NSW and to stillbirths and prenatal therapeutic abortion occurring preferentially in CA babies implies that the present findings are conservative estimates. The very high CA rate reported in Queensland has not been explained despite formal enquiry. The origin of the NNSW denominator figure is unclear. NSW Mothers and Babies reports [7] indicate that during 2008–2015 22,084 babies were born in Northern NSW and 30,848 in the central coast region, totalling 52,932 births. These regions are shown together in our maps. Hence over 11 times the data is available as was used in this analysis if it can be properly collated between the two jurisdictions of NSW and Queensland. This would then facilitate geotemporospatial statistical modelling. This proper collation and assembly of data is a top research priority for future studies. The remote location of NNSW together with its somewhat trans-jurisdictional status has apparently made such a collation difficult in the past.

## Conclusions

In conclusion study data indicate that prenatal cannabis exposure is a significant and robust covariate of many congenital anomalies in NNSW particularly affecting the cardiovascular, chromosomal, body wall and gastrointestinal systems and is highly significant for 10 cannabis-related defects on geospatial analysis. Close concordance between these results and previous reports from Hawaii, Colorado, and Canada and with unpublished USA studies suggest our findings are reliable and generalizable. On all studies cannabis teratogenesis seems to be more concerning than the established teratogens tobacco and alcohol. Fulfillment of the criteria for causal relationships has been demonstrated. Further geospatial epidemiological and basic science research is a priority given cannabis commercialization. Even beyond the obvious jurisdictional health cost-shifting implications careful and thorough further investigation of the teratological profile of NNSW by coordinated investigations between NSW and Queensland over time to current would appear to be a major international research priority with implications far beyond our shores.

## Supplementary information

**Additional file 1: Table 1** Input Data – Rates and Numbers. **Table 2** Drug Use by Area Data – Mean NDSHS 2010, 2013. **Table 3** Significant of rises of Supplementary Fig. 1. **Table 4** 2 × 2 Table Analysis Output by Defect and Defect Class. **Table 5** Regression Coefficients and Significance Levels for Defects against Tobacco, Alcohol and Cannabis Use Ordered by Significance of Regression on Cannabis . **Table 6** Regression Coefficients and Significance Levels for Defects against Tobacco, Alcohol and Cannabis Use Ordered by Significance of Regression on Tobacco. **Table 7** Regression Coefficients and Significance Levels for Defects against Tobacco, Alcohol and Cannabis Use Ordered by Significance of Regression on Alcohol. **Table 8** Expanded Outcomes from Additive Linear Modelling of 3 Drug Exposure – *P* < 0.3. **Table 9** Linear Interactive Final Models.

**Additional file 2 Supplementary Fig. 1.:** Choropleth maps of congenital anomaly class rates across QLD and NNSW for Congenital anopmalies A-C. High rates are shown in yellow and low rates in dark blue. Maps were drawn using R package “sf” [15]. **Supplementary Fig. 2.:** Choropleth maps of congenital anomaly class rates across QLD and NNSW for Congenital anopmalies C-P. High rates are shown in yellow and low rates in dark blue. Maps were drawn using R package “sf” [15]. **Supplementary Fig. 3.:** Choropleth maps of congenital anomaly class rates across QLD and NNSW for Congenital anopmalies R-Z. High rates are shown in yellow and low rates in dark blue. Maps were drawn using R package “sf” [15].

**Additional file 3.**

## Data Availability

All data generated or analysed during this study are included in this published article and its supplementary information files. Data has been made publicly available on the Mendeley Database Repository and can be accessed from this URL 10.17632/cjzfyktz5m.1.

## Abbreviations

AAP: American Academy of Pediatrics
ACD: All Chromsomal Defects
ACT: Anal stenosis, Congenital hydrocephalus and Turner syndrome
AFE: Attributable Fraction in the Exposed
AFP: Attributable Fraction in the Population
AHA: American Heart Association
ASD: Atrial Septal Defect
AVTPCDSGDH: Atrial septal defect, Ventricular septal defect, Tetralogy of Fallot, Patent ductus arteriosus, Chromosomal defects, Downs syndrome, Small intestinal atresia, Gastroschisis, Diaphragmatic hernia, Hypospadias
C.I.: Confidence Interval
CA: Congenital Anomalies
CALF: Congential Anomaly Linked File in Queensland Health [6]
CDC: Center for Disease Control
CNRD: Cannabis Non-Related defects
CNS: Central Nervous System
CRAN: Central “R” Archive Network
CRD: Cannabis Related defects
CVS: Cardiovascular System
E-Values: Expected Values
IPW: Inverse Probability Weighting
KKP: Kapoor, Kelejian, and Prucha [17]
NDSHS: National Drug Strategy Household Survey
NNSW: Northern New South Wales
NRS: Nepnatal Retrieval System
NSW: New South Wales
PCE: Prenatal Cannabis Exposure
PDA: Patent Ductus Arteriosus
PR: Prevalence Ratio
QLD: Queensland
SARAR: Spatial Auto-Regressive Models with Auto-Regressive Error Terms
SEM: Spatial Error Model
sf: Simple Features
ToF: Tetralogy of Fallot
TxGrVess: Transpostion of the Great Vessels
VSD: Ventricular Septal Defect

## References

[1] Potential Big Tobacco Acquisition Causes Cannabis Company's Stock to Soar [https://www.forbes.com/sites/sarabrittanysomerset/2018/12/04/possible-big-tobacco-deal-causes-cronos-stock-to-soar/#48e3a6cc4891]. Accessed 6 Oct 2020.

[2] Reece AS, Hulse GK. Canadian cannabis consumption and patterns of congenital anomalies: an ecological geospatial analysis. J Addict Med. 2020;14(5):e195–e210. 10.1097/ADM.0000000000000638. 10.1097/ADM.0000000000000638 PMC7547880 32187114

[3] Reece AS, Hulse GK (2019). Cannabis teratology explains current patterns of Coloradan congenital defects: the contribution of increased cannabinoid exposure to rising Teratological trends. Clin Pediatr.

[4] Forrester MB, Merz RD (2007). Risk of selected birth defects with prenatal illicit drug use, Hawaii, 1986-2002. J Toxicol Environ Health.

[5] Neonatal Retrieval Service (NeoRESQ) [https://metronorth.health.qld.gov.au/rbwh/healthcare-services/neonatal-retrieval-service-neoresq]. Accessed 6 Oct 2020.

[6] Report of the Queensland Perinatal Maternal and Perinatal Quality Council, Queensland Health. Congenital anomaly linked file (CALF): data table and notes, 2017. In*.* Edited by Queensland Health, vol. 1. Brisbane: Queensland Health; 2018. p. 5. https://www.health.qld.gov.au/hsu/dashboards.

[7] New South Wales Health Department (2018). NSW mothers and babies 2018.

[8] Queensland Maternal and Perinatal Quality Council 2017: Queensland mothers and babies 2014 and 2015. In*.* Edited by health Q, vol. 1. Brisbane: Queensland Health; 2018: 1–70.

[9] Australian Institute of Health and Welfare: National Drug Strategy Household Survey 2016: detailed findings. In*.* Edited by Australian Institute of Health and Welfare, vol. 1. Canberra: Australian Institute of Health and Welfare,; 2017: 168.

[10] PHN Digital Files [https://www1.health.gov.au/internet/main/publishing.nsf/Content/PHN-Digital]. Accessed 6 Oct 2020.

[11] Reece AS, Hulse GK. Cannabis consumption patterns parallel the east-west gradient in Canadian neural tube defect incidence: an ecological study. Global Pediatric Health. 2019;6:1–12. 10.1177/2333794X19894798 PMC6906350 31853464

[12] Reece AS, Hulse GK. Impacts of cannabinoid epigenetics on human development: reflections on murphy et. al. 'Cannabinoid exposure and altered DNA methylation in rat and human Sperm' epigenetics. Epigenetics. 2019;14(11):1041–56.10.1080/15592294.2019.1633868. 10.1080/15592294.2019.1633868 PMC6773386 31293213

[13] Reece AS. Chronic toxicology of cannabis. Clin Toxicol. 2009;47(6):517–24. 10.1080/15563650903074507 19586351

[14] Wickham H (2016). ggplot2: elegant graphics for data analysis, vol. 1.

[15] Pebesma E (2018). Simple features for R: standardized support for spatial vector data. R J.

[16] Millo G, Piras G (2012). Splm: spatial panel data models in R. J Stastistical Software.

[17] Kapoor M, Kelejian HH, Prucha IR (2007). Panel data models with spatially correlated error components. J Econ.

[18] Croissant Y, Millo G (2019). Panel data econometrics with R, vol. 1.

[19] Jenkins KJ, Correa A, Feinstein JA, Botto L, Britt AE, Daniels SR (2007). Noninherited risk factors and congenital cardiovascular defects: current knowledge: a scientific statement from the American Heart Association Council on cardiovascular disease in the young: endorsed by the American Academy of Pediatrics. Circulation.

[20] Van Gelder MMHJ, Donders ART, Devine O, Roeleveld N, Reefhuis J (2014). Using bayesian models to assess the effects of under-reporting of cannabis use on the association with birth defects, national birth defects prevention study, 1997-2005. Paediatr Perinat Epidemiol.

[21] VanderWeele TJ, Ding P, Mathur M (2019). Technical considerations in the use of the E-value. J Causal Inference.

[22] Alshehri A, Emil S, Laberge JM, Skarsgard E (2013). Canadian pediatric surgery N: outcomes of early versus late intestinal operations in patients with gastroschisis and intestinal atresia: results from a prospective national database. J Pediatr Surg.

[23] Geber WF, Schramm LC (1969). Effect of marihuana extract on fetal hamsters and rabbits. Toxicol Appl Pharmacol.

[24] Graham JDP, Graham JDP (1976). Cannabis and health. Cannabis and health. Volume 1.

[25] Endo T., Johnston T., Ellerington J., Donovan T.: Gastroschisis in Queensland. In*.* Edited by Health Statistics Unit, Queensland Health, GPO Box 48, Brisbane Q, Australia 4001., vol. StatBite #57. Brisbane: Queensland Health; 2013.

[26] Wilson PD, Loffredo CA, Correa-Villasenor A, Ferencz C (1998). Attributable fraction for cardiac malformations. Am J Epidemiol.

[27] David AL, Holloway A, Thomasson L, Syngelaki A, Nicolaides K, Patel RR (2014). A case-control study of maternal periconceptual and pregnancy recreational drug use and fetal malformation using hair analysis. PLoS One.

[28] Draper ES, Rankin J, Tonks AM, Abrams KR, Field DJ, Clarke M (2008). Recreational drug use: a major risk factor for gastroschisis?. Am J Epidemiol.

[29] Skarsgard ED, Meaney C, Bassil K, Brindle M, Arbour L, Moineddin R (2015). Maternal risk factors for gastroschisis in Canada. Birth Defects Res A Clin Mol Teratol.

[30] Torfs CP, Velie EM, Oechsli FW, Bateson TF, Curry CJ (1994). A population-based study of gastroschisis: demographic, pregnancy, and lifestyle risk factors. Teratology.

[31] van Gelder MM, Reefhuis J, Caton AR, Werler MM, Druschel CM, Roeleveld N (2009). National Birth Defects Prevention S: maternal periconceptional illicit drug use and the risk of congenital malformations. Epidemiology.

[32] Werler MM, Sheehan JE, Mitchell AA (2003). Association of vasoconstrictive exposures with risks of gastroschisis and small intestinal atresia. Epidemiology.

[33] Expert Review Panel Appointed to New South Wales Health: Review of Gastroschisis on the NSW north coast. In*.* Edited by From New South Wales Health Department Center for Record Linkage DC, Dr Lee Taylor, ltayl@doh.health.nsw.gov.au ,, vol. 1. Sydney: New South Wales Health Department; 2011: 1–11.

[34] Reece AS, Wang W, Hulse GK (2018). Pathways from epigenomics and glycobiology towards novel biomarkers of addiction and its radical cure. Med Hypotheses.

[35] Szutorisz H, DiNieri JA, Sweet E, Egervari G, Michaelides M, Carter JM (2014). Parental THC exposure leads to compulsive heroin-seeking and altered striatal synaptic plasticity in the subsequent generation. Neuropsychopharmacology.

[36] Watson CT, Szutorisz H, Garg P, Martin Q, Landry JA, Sharp AJ (2015). Genome-wide DNA methylation profiling reveals epigenetic changes in the rat nucleus Accumbens associated with cross-generational effects of adolescent THC exposure. Neuropsychopharmacology.

[37] Reece AS, Hulse GK (2016). Chromothripsis and epigenomics complete causality criteria for cannabis- and addiction-connected carcinogenicity, congenital toxicity and heritable genotoxicity. Mutat Res.

[38] Reece AS, Hulse GK (2019). Effect of cannabis legalization on US autism incidence and medium term projections. Clin Pediatr.

[39] Reece AS, Hulse GK (2019). Epidemiological associations of various substances and multiple cannabinoids with autism in USA. Clin Pediatr.

[40] Brents L, Preedy VR, Correlates and consequences of Prenatal Cannabis Exposure (PCE) (2017). Identifying and characterizing vulnerable maternal populations and determining outcomes in exposed offspring. Handbook of cannabis and related pathologies: biology, pharmacology, diagnosis and treatment. Volume 1.

[41] Smith AM, Mioduszewski O, Hatchard T, Byron-Alhassan A, Fall C, Fried PA (2016). Prenatal marijuana exposure impacts executive functioning into young adulthood: an fMRI study. Neurotoxicol Teratol.

[42] Smith AM, Longo CA, Fried PA, Hogan MJ, Cameron I (2010). Effects of marijuana on visuospatial working memory: an fMRI study in young adults. Psychopharmacology.

[43] Reece AS, Hulse GK (2019). Gastroschisis and autism-dual canaries in the Californian coalmine. JAMA Surg.

[44] Ordean A, Wong S, Graves L (2017). No. 349-substance use in pregnancy. J Obstet Gynaecol Can.

[45] Pearl J, Mackenzie D (2018). The book of why. The new science of cause and effect vol. 1.

[46] Causal Inference in Statistics: A Gentle Introduction [http://bayes.cs.ucla.edu/jp_home.html;http://bayes.cs.ucla.edu/jsm-august2016-bw.pdf]. Accessed 6 Oct 2020.

[47] Szutorisz H, Egervari G, Sperry J, Carter JM, Hurd YL (2016). Cross-generational THC exposure alters the developmental sensitivity of ventral and dorsal striatal gene expression in male and female offspring. Neurotoxicol Teratol.

[48] Szutorisz H, Hurd YL (2016). Epigenetic effects of cannabis exposure. Biol Psychiatry.

[49] Szutorisz H, Hurd YL (2018). High times for cannabis: epigenetic imprint and its legacy on brain and behavior. Neurosci Biobehav Rev.

[50] DiNieri JA, Wang X, Szutorisz H, Spano SM, Kaur J, Casaccia P (2011). Maternal cannabis use alters ventral striatal dopamine D2 gene regulation in the offspring. Biol Psychiatry.

[51] Hill AB (1965). The environment and disease: association or causation?. Proc R Soc Med.

