# Supplementary material for: Broad Spectrum epidemiological contribution of cannabis and other substances to the teratological profile of northern New South Wales: geospatial and causal inference analysis
Source: BMC Pharmacol Toxicol. 2020 Nov 12;21:75. doi: 10.1186/s40360-020-00450-1 (PMC7659114; doi:10.1186/s40360-020-00450-1)
Supplement: Supplementary file 1 — Additional file 1: Table 1 Input Data – Rates and Numbers. Table 2 Drug Use by Area Data – Mean NDSHS 2010, 2013. Table 3 Significant of rises of Supplementary Fig. 1. Table 4 2 × 2 Table Analysis Output by Defect and Defect Class. Table 5 Regression Coefficients and Significance Levels for Defects against Tobacco, Alcohol and Cannabis Use Ordered by Significance of Regression on Cannabis . Table 6 Regression Coefficients and Significance Levels for Defects against Tobacco, Alcohol and Cannabis Use Ordered by Significance of Regression on Tobacco. Table 7 Regression Coefficients and Significance Levels for Defects against Tobacco, Alcohol and Cannabis Use Ordered by Significance of Regression on Alcohol. Table 8 Expanded Outcomes from Additive Linear Modelling of 3 Drug Exposure – P < 0.3. Table 9 Linear Interactive Final Models. [file 40360_2020_450_MOESM1_ESM.docx]

**Supplementary Tables**

**Table of Contents**

| **Item** | **Name** | **Page** |
| --- | --- | --- |
|  |  |  |
| ***Supplementary Tables*** |  |  |
| Table 1 | Input Data – Rates and Numbers | 2 |
| Table 2 | Drug Use by Area Data – Mean NDSHS 2010, 2013 | 7 |
| Table 3 | Significant of rises of Supplementary Figure 1 | 8 |
| Table 4 | 2x2 Table Analysis Output by Defect and Defect Class | 9 |
| Table 5 | Regression Coefficients and Significance Levels for Defects against Tobacco, Alcohol and Cannabis Use Ordered by Significance of Regression on Cannabis | 14 |
| Table 6 | Regression Coefficients and Significance Levels for Defects against Tobacco, Alcohol and Cannabis Use Ordered by Significance of Regression on Tobacco | 17 |
| Table 7 | Regression Coefficients and Significance Levels for Defects against Tobacco, Alcohol and Cannabis Use Ordered by Significance of Regression on Alcohol | 20 |
| Table 8 | Expanded Outcomes from Additive Linear Modelling of 3 Drug Exposure – P<0.3 | 24 |
| Table 9 | Linear Interactive Final Models | 26 |
|  |  |  |

**Supplementary Table 1.: Input Data – Rates and Numbers**

| Defect | Relationship to Cannabis | Interstate Rate | QLD Rate | Differ-ence | Ratio | Interstate Numbers | Normal Interstate Births | QLD Numbers | Normal QLD  Births |
| --- | --- | --- | --- | --- | --- | --- | --- | --- | --- |
|  |  |  |  |  |  |  |  |  |  |
| ***Defect*** |  |  |  |  |  |  |  |  |  |
| Exomphalos | Related | 1.5 | 0.2 | 1.3 | 7.50 | 7 | 4793 | 118 | 508970 |
| Gastroschisis | Related | 3.3 | 0.6 | 2.7 | 5.50 | 16 | 4784 | 290 | 508789 |
| Trisomy 18 | Related | 2.3 | 0.5 | 1.8 | 4.60 | 11 | 4789 | 259 | 508825 |
| Stenosis/atresia small intestine | Related | 1.3 | 0.3 | 1 | 4.33 | 6 | 4794 | 161 | 508928 |
| Diaphragmatic hernia | Related | 1.3 | 0.3 | 1 | 4.33 | 6 | 4794 | 144 | 508945 |
| Tetralogy of Fallot | Related | 1.7 | 0.4 | 1.3 | 4.25 | 8 | 4792 | 185 | 508902 |
| Transposition of great vessels | Related | 2.5 | 0.6 | 1.9 | 4.17 | 12 | 4788 | 274 | 508809 |
| Turner syndrome | Related | 0.8 | 0.2 | 0.6 | 4.00 | 4 | 4796 | 115 | 508976 |
| Patent Ductus Arteriosus | Related | 21.3 | 6.5 | 14.8 | 3.28 | 102 | 4698 | 3149 | 505844 |
| Atrial Septal Defects | Related | 16.9 | 5.4 | 11.5 | 3.13 | 81 | 4719 | 2624 | 506390 |
| Ventricular Septal Defect | Related | 10.8 | 4.7 | 6.1 | 2.30 | 52 | 4748 | 2323 | 506720 |
| Anencephalus | Related | 1 | 0.5 | 0.5 | 2.00 | 5 | 4795 | 242 | 508848 |
| Encephalocoele | Related | 0.2 | 0.1 | 0.1 | 2.00 | 1 | 4799 | 47 | 509047 |
| Anotia, microtia | Related | 0.4 | 0.2 | 0.2 | 2.00 | 2 | 4798 | 110 | 508983 |
| Tracheo-oesophageal fistula, oesophageal atresia/s | Related | 0.6 | 0.3 | 0.3 | 2.00 | 3 | 4797 | 172 | 508920 |
| Trisomy 13 | Related | 0.4 | 0.2 | 0.2 | 2.00 | 2 | 4798 | 97 | 508996 |
| Reduction deformities Upper and/or lower limbs | Related | 1.3 | 0.7 | 0.6 | 1.86 | 6 | 4794 | 329 | 508760 |
| Neural tube defects | Related | 2.3 | 1.3 | 1 | 1.77 | 11 | 4789 | 623 | 508461 |
| Down syndrome | Related | 3.5 | 2 | 1.5 | 1.75 | 17 | 4783 | 987 | 508091 |
| Spina Bifida | Related | 1 | 0.6 | 0.4 | 1.67 | 5 | 4795 | 274 | 508816 |
| Renal agenesis or dysgenesis | Related | 2.1 | 1.3 | 0.8 | 1.62 | 10 | 4790 | 657 | 508428 |
| Microcephaly | Related | 0.8 | 0.5 | 0.3 | 1.60 | 4 | 4796 | 263 | 508828 |
| Cleft Palate/Cleft lip | Related | 2.5 | 1.9 | 0.6 | 1.32 | 12 | 4788 | 959 | 508124 |
| Stenosis/atresia anus | Related | 0.4 | 0.4 | 0 | 1.00 | 2 | 4798 | 202 | 508891 |
| Syndactyly | Related | 1 | 1 | 0 | 1.00 | 5 | 4795 | 496 | 508594 |
| Polydactyly | Related | 0.4 | 1.2 | -0.8 | 0.33 | 2 | 4798 | 589 | 508504 |
| Pyloric Stenosis | Related | 0.4 | 1.3 | -0.9 | 0.31 | 2 | 4798 | 657 | 508436 |
| Cystic Kidney disease | Not Related | 1 | 0.3 | 0.7 | 3.33 | 5 | 4795 | 159 | 508931 |
| Congenital hydrocephalus (excl. those with NTD) | Not Related | 1.9 | 0.6 | 1.3 | 3.17 | 9 | 4791 | 292 | 508794 |
| Coarctation of Aorta | Not Related | 1.9 | 0.6 | 1.3 | 3.17 | 9 | 4791 | 284 | 508802 |
| Choanal Atresia | Not Related | 0.6 | 0.2 | 0.4 | 3.00 | 3 | 4797 | 122 | 508970 |
| Microphthalmia | Not Related | 0.2 | 0.1 | 0.1 | 2.00 | 1 | 4799 | 48 | 509046 |
| Disorders of amino acid transport and metabolism | Not Related | 0.4 | 0.2 | 0.2 | 2.00 | 2 | 4798 | 122 | 508971 |
| Disorders of carbohydrate transport and metabolism | Not Related | 0.2 | 0.1 | 0.1 | 2.00 | 1 | 4799 | 53 | 509041 |
| Branchial Remnants | Not Related | 0.8 | 0.5 | 0.3 | 1.60 | 4 | 4796 | 245 | 508846 |
| Developmental dysplasia of hip | Not Related | 5.6 | 3.8 | 1.8 | 1.47 | 27 | 4773 | 1864 | 507204 |
| Congenital cataract and lens anomalies | Not Related | 0.4 | 0.3 | 0.1 | 1.33 | 2 | 4798 | 159 | 508934 |
| Undescended Testis (treated) | Not Related | 8.8 | 8.8 | 0 | 1.00 | 42 | 4758 | 4371 | 504682 |
| Craniosynostosis | Not Related | 1 | 1 | 0 | 1.00 | 5 | 4795 | 479 | 508611 |
| Obstructive defects renal pelvis | Not Related | 2.3 | 2.8 | -0.5 | 0.82 | 11 | 4789 | 1375 | 507709 |
| Hypospadias | Not Related | 2.7 | 4.1 | -1.4 | 0.66 | 13 | 4787 | 2020 | 507062 |
| Talipes | Not Related | 8.5 | 16.1 | -7.6 | 0.53 | 41 | 4759 | 8027 | 501027 |
| Other anomalies of ureter | Not Related | 0.2 | 0.5 | -0.3 | 0.40 | 1 | 4799 | 259 | 508835 |
| Birth marks, nevus | Not Related | 1.9 | 7.7 | -5.8 | 0.25 | 9 | 4791 | 3858 | 505228 |
|  |  |  |  |  |  |  |  |  |  |
| ***Defect Class*** |  |  |  |  |  |  |  |  |  |
| CHROMOSOMAL DEFECTS | Related | 9.8 | 4.5 | 5.3 | 2.18 | 47 | 4753 | 2191 | 506857 |
| CARDIOVASCULAR DEFECTS | Related | 38.1 | 17.8 | 20.3 | 2.14 | 183 | 4617 | 8747 | 500165 |
| NERVOUS SYSTEM DEFECTS | Related | 7.5 | 4.2 | 3.3 | 1.79 | 36 | 4764 | 2051 | 507008 |
| CONGENITAL ANOMALIES OF EAR, FACE AND NECK | Related | 4.2 | 3.2 | 1 | 1.31 | 20 | 4780 | 1610 | 507465 |
| GASTRO-INTESTINAL DEFECTS | Related | 22.3 | 26.9 | -4.6 | 0.83 | 107 | 4693 | 13414 | 495574 |
| RESPIRATORY SYSTEM DEFECTS | Not Related | 7.5 | 4.7 | 2.8 | 1.60 | 36 | 4764 | 2332 | 506727 |
| CONGENITAL ANOMALIES OF EYE | Not Related | 2.3 | 2 | 0.3 | 1.15 | 11 | 4789 | 992 | 508092 |
| URI-GENITAL DEFECTS | Not Related | 18.3 | 20.4 | -2.1 | 0.90 | 88 | 4712 | 10156 | 498851 |
| MUSCULO-SKELETAL DEFECTS | Not Related | 32.3 | 38.2 | -5.9 | 0.85 | 155 | 4645 | 19007 | 489933 |
| CONGENITAL ANOMALIES OF INTEGUMENT | Not Related | 7.1 | 17.2 | -10.1 | 0.41 | 34 | 4766 | 8603 | 500458 |

| Defect | Interstate Lower C.I. | Interstate Upper C.I. | QLD Lower C.I. | QLD Upper C.I. |
| --- | --- | --- | --- | --- |
|  |  |  |  |  |
| ***Defect*** |  |  |  |  |
| Exomphalos | 0.7 | 3 | 0.2 | 0.3 |
| Gastroschisis | 2.1 | 5.4 | 0.5 | 0.7 |
| Trisomy 18 | 1.3 | 4.1 | 0.5 | 0.6 |
| Stenosis/atresia small intestine | 0.6 | 2.7 | 0.3 | 0.4 |
| Diaphragmatic hernia | 0.6 | 2.7 | 0.3 | 0.4 |
| Tetralogy of Fallot | 0.8 | 3.3 | 0.3 | 0.4 |
| Transposition of great vessels | 1.4 | 4.4 | 0.5 | 0.6 |
| Turner syndrome | 0.3 | 2.1 | 0.2 | 0.3 |
| Patent Ductus Arteriosus | 17.5 | 25.7 | 6.3 | 6.7 |
| Atrial Septal Defects | 13.6 | 20.9 | 5.2 | 5.6 |
| Ventricular Septal Defect | 8.3 | 14.2 | 4.5 | 4.9 |
| Anencephalus | 0.4 | 2.4 | 0.4 | 0.6 |
| Encephalocoele | 0 | 1.2 | 0.1 | 0.1 |
| Anotia, microtia | 0.1 | 1.5 | 0.2 | 0.3 |
| Tracheo-oesophageal fistula, oesophageal atresia/s | 0.2 | 1.8 | 0.3 | 0.4 |
| Trisomy 13 | 0.1 | 1.5 | 0.2 | 0.2 |
| Reduction deformities Upper and/or lower limbs | 0.6 | 2.7 | 0.6 | 0.7 |
| Neural tube defects | 1.3 | 4.1 | 1.2 | 1.4 |
| Down syndrome | 2.2 | 5.7 | 1.9 | 2.1 |
| Spina Bifida | 0.4 | 2.4 | 0.5 | 0.6 |
| Renal agenesis or dysgenesis | 1.1 | 3.8 | 1.2 | 1.4 |
| Microcephaly | 0.3 | 2.1 | 0.5 | 0.6 |
| Cleft Palate/Cleft lip | 1.4 | 4.4 | 1.8 | 2.1 |
| Stenosis/atresia anus | 0.1 | 1.5 | 0.4 | 0.5 |
| Syndactyly | 0.4 | 2.4 | 0.9 | 1.1 |
| Polydactyly | 0.1 | 1.5 | 1.1 | 1.3 |
| Pyloric Stenosis | 0.1 | 1.5 | 1.2 | 1.4 |
| Cystic Kidney disease | 0.4 | 2.4 | 0.3 | 0.4 |
| Congenital hydrocephalus (excl. those with NTD) | 1 | 3.6 | 0.5 | 0.7 |
| Coarctation of Aorta | 1 | 3.6 | 0.5 | 0.7 |
| Choanal Atresia | 0.2 | 1.8 | 0.2 | 0.3 |
| Microphthalmia | 0 | 1.2 | 0.1 | 0.1 |
| Disorders of amino acid transport and metabolism | 0.1 | 1.5 | 0.2 | 0.3 |
| Disorders of carbohydrate transport and metabolism | 0 | 1.2 | 0.1 | 0.1 |
| Branchial Remnants | 0.3 | 2.1 | 0.4 | 0.6 |
| Developmental dysplasia of hip | 3.9 | 8.2 | 3.6 | 3.9 |
| Congenital cataract and lens anomalies | 0.1 | 1.5 | 0.3 | 0.4 |
| Undescended Testis (treated) | 6.5 | 11.8 | 8.5 | 9.1 |
| Craniosynostosis | 0.4 | 2.4 | 0.9 | 1.1 |
| Obstructive defects renal pelvis | 1.3 | 4.1 | 2.6 | 2.9 |
| Hypospadias | 1.6 | 4.6 | 3.9 | 4.2 |
| Talipes | 6.3 | 11.6 | 15.7 | 16.4 |
| Other anomalies of ureter | 0 | 1.2 | 0.5 | 0.6 |
| Birth marks, nevus | 1 | 3.6 | 7.5 | 8 |
|  |  |  |  |  |
| ***Defect Class*** |  |  |  |  |
| CHROMOSOMAL DEFECTS | 7.4 | 13 | 4.3 | 4.6 |
| CARDIOVASCULAR DEFECTS | 33.1 | 43.9 | 17.4 | 18.2 |
| NERVOUS SYSTEM DEFECTS | 5.4 | 10.4 | 4 | 4.3 |
| CONGENITAL ANOMALIES OF EAR, FACE AND NECK | 2.7 | 6.4 | 3.1 | 3.4 |
| GASTRO-INTESTINAL DEFECTS | 18.5 | 26.9 | 26.5 | 27.4 |
| RESPIRATORY SYSTEM DEFECTS | 5.4 | 10.4 | 4.5 | 4.9 |
| CONGENITAL ANOMALIES OF EYE | 1.3 | 4.1 | 1.9 | 2.1 |
| URO-GENITAL DEFECTS | 14.9 | 22.5 | 20 | 20.8 |
| MUSCULO-SKELETAL DEFECTS | 27.7 | 37.7 | 37.7 | 38.7 |
| CONGENITAL ANOMALIES OF INTEGUMENT | 5.1 | 9.9 | 16.9 | 17.6 |

**Supplementary Table 2.: Drug Use by Area Data – Mean NDSHS 2010, 2013**

| **Area** | **Daily Cigarette Use** | **Risk Alcohol Use** | **Cannabis Use, Annual** | **Tobacco Ranking** | **Binge Alcohol Ranking** | **Cannabis Ranking** |
| --- | --- | --- | --- | --- | --- | --- |
|  |  |  |  |  |  |  |
| Richmond.Tweed | 15.5 | 29.1 | 18.6 | 11 | 13 | 1 |
| Cairns | 20.15 | 29.45 | 16.5 | 4 | 11 | 2 |
| Brisbane Inner City | 9.5 | 36.75 | 16.1 | 19 | 4 | 3 |
| Townsville | 22.85 | 42.35 | 13.9 | 2 | 1 | 4 |
| Gold Coast | 14.5 | 33 | 12.3 | 14 | 7 | 5 |
| Brisbane.South | 10.55 | 28.05 | 11.4 | 18 | 14 | 6 |
| Sunshine Coast | 12.5 | 29.15 | 11.2 | 16 | 12 | 7 |
| Wide Bay | 23.55 | 27.65 | 11.2 | 1 | 16 | 8 |
| Brisbane.East | 12.4 | 33 | 11.0 | 17 | 8 | 9 |
| Central Queensland | 19.65 | 39.9 | 10.9 | 6 | 2 | 10 |
| Central Queensland | 19.65 | 39.9 | 10.9 | 7 | 3 | 11 |
| Logan.Beaudesert | 20.05 | 27.65 | 10.5 | 5 | 17 | 12 |
| Australia | 13.95 | 28 | 10.3 | 15 | 15 | 13 |
| Moreton Bay.South | 15.85 | 33.85 | 10.0 | 10 | 5 | 14 |
| Brisbane.West | 7.45 | 30.8 | 9.3 | 21 | 9 | 15 |
| Ipswich | 18.9 | 23.25 | 8.8 | 8 | 21 | 16 |
| Moreton Bay.North | 17.25 | 30.55 | 8.6 | 9 | 10 | 17 |
| Toowoomba | 14.65 | 26.75 | 7.0 | 13 | 19 | 18 |
| Queensland.Outback | 15.5 | 33.6 | 6.2 | 12 | 6 | 19 |
| Darling Downs.Maranoa | 20.85 | 24.85 | 5.6 | 3 | 20 | 20 |
| Brisbane.North | 8.95 | 26.8 | 5.5 | 20 | 18 | 21 |

In this Table Richmond Tweed refers to the Northern New South Wales Area.

**Supplementary Table 3.: Significant of rises of Supplementary Figure 1**

| **Anomaly / Group** | **Chi Squared** | **df** | **P** |
| --- | --- | --- | --- |
|  |  |  |  |
| Cardiovascular Defects | 96.415 | 7 | 9.31E-23 |
| Atrial septal Defect | 70.943 | 7 | 9.53E-13 |
| Ventricular Septal Defect | 29.162 | 7 | 1.52E-04 |
| Patent Ductus Arteriosus | 23.755 | 7 | 1.26E-03 |
| Respiratory System Defects | 149.840 | 7 | 1.88E-34 |
| Gastrointestinal System Defects | 1210.600 | 7 | 3.03E-265 |

**Supplementary Table 4.: 2x2 Table Analysis Output by Defect and Defect Class**

| Defect | CanRel | Interstate Numbers | Normal Interstate Births | QLD Numbers | Normal QLD  Births | PR | PR_LCI | PR_UCI |
| --- | --- | --- | --- | --- | --- | --- | --- | --- |
|  |  |  |  |  |  |  |  |  |
| ***Defect*** |  |  |  |  |  |  |  |  |
| Exomphalos | Related | 7 | 4793 | 118 | 508970 | 6.29 | 2.94 | 13.48 |
| Gastroschisis | Related | 16 | 4784 | 290 | 508789 | 5.85 | 3.54 | 9.67 |
| Trisomy 18 | Related | 11 | 4789 | 259 | 508825 | 4.50 | 2.46 | 8.23 |
| Stenosis/atresia small intestine | Related | 6 | 4794 | 161 | 508928 | 3.95 | 1.75 | 8.92 |
| Diaphragmatic hernia | Related | 6 | 4794 | 144 | 508945 | 4.42 | 1.95 | 10.00 |
| Tetralogy of Fallot | Related | 8 | 4792 | 185 | 508902 | 4.59 | 2.26 | 9.30 |
| Transposition of great vessels | Related | 12 | 4788 | 274 | 508809 | 4.65 | 2.61 | 8.28 |
| Turner syndrome | Related | 4 | 4796 | 115 | 508976 | 3.69 | 1.36 | 9.99 |
| Patent Ductus Arteriosus | Related | 102 | 4698 | 3149 | 505844 | 3.43 | 2.84 | 4.17 |
| Atrial Septal Defects | Related | 81 | 4719 | 2624 | 506390 | 3.27 | 2.63 | 4.08 |
| Ventricular Septal Defect | Related | 52 | 4748 | 2323 | 506720 | 2.37 | 1.81 | 3.12 |
| Anencephalus | Related | 5 | 4795 | 242 | 508848 | 2.19 | 0.90 | 5.31 |
| Encephalocoele | Related | 1 | 4799 | 47 | 509047 | 2.26 | 0.31 | 16.36 |
| Anotia, microtia | Related | 2 | 4798 | 110 | 508983 | 1.93 | 0.48 | 7.80 |
| Tracheo-oesophageal fistula, oesophageal atresia/s | Related | 3 | 4797 | 172 | 508920 | 1.85 | 0.59 | 5.79 |
| Trisomy 13 | Related | 2 | 4798 | 97 | 508996 | 2.19 | 0.54 | 8.87 |
| Reduction deformities Upper and/or lower limbs | Related | 6 | 4794 | 329 | 508760 | 1.93 | 0.86 | 4.33 |
| Neural tube defects | Related | 11 | 4789 | 623 | 508461 | 1.87 | 1.03 | 3.39 |
| Down syndrome | Related | 17 | 4783 | 987 | 508091 | 1.83 | 1.13 | 2.95 |
| Spina Bifida | Related | 5 | 4795 | 274 | 508816 | 1.94 | 0.80 | 4.68 |
| Renal agenesis or dysgenesis | Related | 10 | 4790 | 657 | 508428 | 1.61 | 0.87 | 3.01 |
| Microcephaly | Related | 4 | 4796 | 263 | 508828 | 1.61 | 0.60 | 4.33 |
| Cleft Palate/Cleft lip | Related | 12 | 4788 | 959 | 508124 | 1.33 | 0.75 | 2.34 |
| Stenosis/atresia anus | Related | 2 | 4798 | 202 | 508891 | 1.05 | 0.26 | 4.23 |
| Syndactyly | Related | 5 | 4795 | 496 | 508594 | 1.07 | 0.44 | 2.58 |
| Polydactyly | Related | 2 | 4798 | 589 | 508504 | 0.36 | 0.09 | 1.44 |
| Pyloric Stenosis | Related | 2 | 4798 | 657 | 508436 | 0.32 | 0.08 | 1.29 |
| Cystic Kidney disease | Not Related | 5 | 4795 | 159 | 508931 | 3.34 | 1.37 | 8.12 |
| Congenital hydrocephalus (excl. those with NTD) | Not Related | 9 | 4791 | 292 | 508794 | 3.27 | 1.69 | 6.34 |
| Coarctation of Aorta | Not Related | 9 | 4791 | 284 | 508802 | 3.36 | 1.73 | 6.52 |
| Choanal Atresia | Not Related | 3 | 4797 | 122 | 508970 | 2.61 | 0.83 | 8.20 |
| Microphthalmia | Not Related | 1 | 4799 | 48 | 509046 | 2.21 | 0.31 | 16.00 |
| Disorders of amino acid transport and metabolism | Not Related | 2 | 4798 | 122 | 508971 | 1.74 | 0.43 | 7.03 |
| Disorders of carbohydrate transport and metabolism | Not Related | 1 | 4799 | 53 | 509041 | 0.20 | 0.03 | 1.42 |
| Branchial Remnants | Not Related | 4 | 4796 | 245 | 508846 | 1.73 | 0.65 | 4.65 |
| Developmental dysplasia of hip | Not Related | 27 | 4773 | 1864 | 507204 | 1.54 | 1.05 | 2.24 |
| Congenital cataract and lens anomalies | Not Related | 2 | 4798 | 159 | 508934 | 1.33 | 0.33 | 5.38 |
| Undescended Testis (treated) | Not Related | 42 | 4758 | 4371 | 504682 | 1.02 | 0.75 | 1.38 |
| Craniosynostosis | Not Related | 5 | 4795 | 479 | 508611 | 1.11 | 0.46 | 2.67 |
| Obstructive defects renal pelvis | Not Related | 11 | 4789 | 1375 | 507709 | 4.01 | 2.20 | 7.32 |
| Hypospadias | Not Related | 13 | 4787 | 2020 | 507062 | 0.68 | 0.40 | 1.18 |
| Talipes | Not Related | 41 | 4759 | 8027 | 501027 | 0.54 | 0.40 | 0.74 |
| Other anomalies of ureter | Not Related | 1 | 4799 | 259 | 508835 | 6.53 | 3.95 | 10.81 |
| Birth marks, nevus | Not Related | 9 | 4791 | 3858 | 505228 | 0.25 | 0.13 | 0.48 |
|  |  |  |  |  |  |  |  |  |
| NOT_CANNABIS_RELATED | Not Related | 185 | 4615 | 23737 | 485358 | 0.83 | 0.72 | 0.95 |
| CANNABIS_RELATED | Related | 394 | 4406 | 16346 | 497155 | 2.56 | 2.32 | 2.81 |
|  |  |  |  |  |  |  |  |  |
| ***Defect Class*** |  |  |  |  |  |  |  |  |
| CHROMOSOMAL DEFECTS | Related | 47 | 4753 | 2191 | 506857 | 2.27 | 1.71 | 3.03 |
| CARDIOVASCULAR DEFECTS | Related | 183 | 4617 | 8747 | 500165 | 2.22 | 1.92 | 2.56 |
| NERVOUS SYSTEM DEFECTS | Related | 36 | 4764 | 2051 | 507008 | 1.86 | 0.34 | 2.58 |
| CONGENITAL ANOMALIES OF EAR, FACE AND NECK | Related | 20 | 4780 | 1610 | 507465 | 1.32 | 0.85 | 2.05 |
| GASTRO-INTESTINAL DEFECTS | Related | 107 | 4693 | 13414 | 495574 | 0.85 | 0.70 | 1.02 |
| RESPIRATORY SYSTEM DEFECTS | Not Related | 36 | 4764 | 2332 | 506727 | 1.64 | 1.18 | 2.27 |
| CONGENITAL ANOMALIES OF EYE | Not Related | 11 | 4789 | 992 | 508092 | 1.18 | 0.65 | 2.13 |
| URO-GENITAL DEFECTS | Not Related | 88 | 4712 | 10156 | 498851 | 0.92 | 0.75 | 1.13 |
| MUSCULO-SKELETAL DEFECTS | Not Related | 155 | 4645 | 19007 | 489933 | 0.86 | 0.74 | 1.01 |
| CONGENITAL ANOMALIES OF INTEGUMENT | Not Related | 34 | 4766 | 8603 | 500458 | 0.85 | 0.70 | 1.02 |
|  |  |  |  |  |  |  |  |  |
| NOT_CANNABIS_RELATED | Not Related | 324 | 4476 | 41090 | 468005 | 0.84 | 0.75 | 0.93 |
| CANNABIS_RELATED | Related | 393 | 4407 | 28013 | 481082 | 1.49 | 1.35 | 1.64 |

| **Defect** | **AFE** | **AFE_LCI** | **AFE_UCI** | **AFP** | **AFP_LCI** | **AFP_UCI** | **P** |
| --- | --- | --- | --- | --- | --- | --- | --- |
|  |  |  |  |  |  |  |  |
| ***Defect*** |  |  |  |  |  |  |  |
| Exomphalos | 84.11 | 65.95 | 92.58 | 4.71 | 0.55 | 8.69 | 2.83E-08 |
| Gastroschisis | 82.91 | 71.75 | 89.66 | 4.34 | 1.79 | 6.82 | 5.62E-15 |
| Trisomy 18 | 77.80 | 59.43 | 87.85 | 3.17 | 0.76 | 5.52 | 8.13E-08 |
| Stenosis/atresia small intestine | 74.70 | 42.88 | 88.79 | 2.68 | -0.21 | 5.49 | 3.53E-04 |
| Diaphragmatic hernia | 77.37 | 48.82 | 90.00 | 3.09 | -0.12 | 6.21 | 9.45E-05 |
| Tetralogy of Fallot | 78.20 | 55.78 | 89.25 | 3.24 | 0.36 | 6.04 | 3.51E-06 |
| Transposition of great vessels | 78.48 | 61.66 | 87.92 | 3.29 | 0.92 | 5.61 | 9.65E-09 |
| Turner syndrome | 72.89 | 26.57 | 89.99 | 2.45 | -0.87 | 5.66 | 5.90E-03 |
| Patent Ductus Arteriosus | 70.89 | 64.61 | 76.05 | 2.22 | 1.62 | 2.82 | 3.30E-39 |
| Atrial Septal Defects | 69.45 | 61.96 | 75.47 | 2.08 | 1.43 | 2.72 | 3.85E-29 |
| Ventricular Septal Defect | 57.88 | 44.63 | 67.95 | 1.27 | 0.67 | 1.86 | 1.83E-10 |
| Anencephalus | 54.37 | -10.58 | 81.17 | 1.10 | -0.69 | 2.86 | 0.0748 |
| Encephalocoele | 55.71 | -220.97 | 93.89 | 1.16 | -3.00 | 5.16 | 0.4075 |
| Anotia, microtia | 48.13 | -109.94 | 87.19 | 0.86 | -1.65 | 3.30 | 0.3488 |
| Tracheo-oesophageal fistula, oesophageal atresia/s | 45.94 | -69.20 | 82.73 | 0.79 | -1.17 | 2.71 | 0.2831 |
| Trisomy 13 | 54.27 | -85.41 | 88.72 | 1.10 | -1.74 | 3.85 | 0.2613 |
| Reduction deformities Upper and/or lower limbs | 48.30 | -15.86 | 76.93 | 0.87 | -0.58 | 2.29 | 0.1028 |
| Neural tube defects | 46.54 | 3.04 | 70.53 | 0.81 | -0.22 | 1.83 | 0.0362 |
| Down syndrome | 45.26 | 11.66 | 66.08 | 0.77 | -0.04 | 1.57 | 0.0123 |
| Spina Bifida | 48.33 | -25.07 | 78.65 | 0.87 | -0.72 | 2.42 | 0.1361 |
| Renal agenesis or dysgenesis | 38.05 | -15.60 | 66.80 | 0.57 | -0.37 | 1.50 | 0.1289 |
| Microcephaly | 38.01 | -66.34 | 76.90 | 0.57 | -0.91 | 2.03 | 0.3377 |
| Cleft Palate/Cleft lip | 24.65 | -33.06 | 57.33 | 0.30 | -0.40 | 1.00 | 0.3279 |
| Stenosis/atresia anus | 4.77 | -283.27 | 76.34 | 0.05 | -1.33 | 1.40 | 0.9436 |
| Syndactyly | 6.47 | -125.60 | 61.22 | 0.06 | -0.82 | 0.94 | 0.8821 |
| Polydactyly | -177.67 | -1012.54 | 30.70 | -0.60 | -1.08 | -0.13 | 0.1320 |
| Pyloric Stenosis | -209.73 | -1140.68 | 22.68 | -0.64 | -1.06 | -0.21 | 0.0925 |
| Cystic Kidney disease | 70.02 | 27.01 | 87.68 | 2.13 | -0.56 | 4.75 | 0.0049 |
| Congenital hydrocephalus (excl. those with NTD) | 69.41 | 40.65 | 84.23 | 2.08 | 0.11 | 4.00 | 2.08E-04 |
| Coarctation of Aorta | 70.25 | 42.06 | 84.67 | 2.16 | 0.14 | 4.13 | 1.42E-04 |
| Choanal Atresia | 61.66 | -20.50 | 87.80 | 1.48 | -1.27 | 4.15 | 0.0884 |
| Microphthalmia | 54.74 | -227.81 | 93.75 | 1.12 | -2.96 | 5.03 | 0.4204 |
| Disorders of amino acid transport and metabolism | 42.49 | -132.52 | 85.77 | 0.69 | -1.58 | 2.90 | 0.4318 |
| Disorders of carbohydrate transport and metabolism | -399.24 | -3449.99 | 29.79 | -0.75 | -1.13 | -0.38 | 0.0741 |
| Branchial Remnants | 42.25 | -55.04 | 78.49 | 0.68 | -0.91 | 2.24 | 0.2699 |
| Developmental dysplasia of hip | 34.91 | 4.92 | 55.43 | 0.50 | -0.04 | 1.04 | 0.0253 |
| Congenital cataract and lens anomalies | 25.04 | -202.24 | 81.41 | 0.31 | -1.43 | 2.02 | 0.6846 |
| Undescended Testis (treated) | 1.87 | -32.80 | 27.49 | 0.02 | -0.27 | 0.31 | 0.9025 |
| Craniosynostosis | 9.67 | -117.90 | 62.56 | 0.10 | -0.81 | 1.00 | 0.8213 |
| Obstructive defects renal pelvis | 75.09 | 54.55 | 86.35 | 2.74 | 0.58 | 4.86 | 9.62E-07 |
| Hypospadias | -46.51 | -152.57 | 15.01 | -0.30 | -0.65 | 0.05 | 0.1664 |
| Talipes | -84.61 | -150.58 | -36.00 | -0.43 | -0.59 | -0.27 | 6.10E-05 |
| Other anomalies of ureter | 84.69 | 74.65 | 90.75 | 4.93 | 2.09 | 7.68 | 4.30E-17 |
| Birth marks, naevus | -301.81 | -672.35 | -109.04 | -0.70 | -0.86 | -0.55 | 5.93E-06 |
|  |  |  |  |  |  |  |  |
| NOT_CANNABIS_RELATED | -20.99 | -39.41 | -4.98 | -0.16 | -0.27 | -0.05 | 0.0081 |
| CANNABIS_RELATED | 60.88 | 56.95 | 64.46 | 1.43 | 1.21 | 1.66 | 6.01E-84 |
|  |  |  |  |  |  |  |  |
| ***Defect Class*** |  |  |  |  |  |  |  |
| CHROMOSOMAL DEFECTS | 56.04 | 41.40 | 67.03 | 1.18 | 0.58 | 1.77 | 9.11E-09 |
| CARDIOVASCULAR DEFECTS | 54.92 | 47.96 | 60.95 | 1.13 | 0.83 | 1.42 | 2.65E-28 |
| NERVOUS SYSTEM DEFECTS | 46.28 | 25.41 | 61.81 | 0.80 | 0.23 | 1.36 | 1.68E-04 |
| CONGENITAL ANOMALIES OF EAR, FACE AND NECK | 24.10 | -17.86 | 51.12 | 0.30 | -0.24 | 0.83 | 0.2182 |
| GASTRO-INTESTINAL DEFECTS | -18.22 | -42.69 | 2.05 | -0.14 | -0.29 | 0.00 | 8.01E-02 |
| RESPIRATORY SYSTEM DEFECTS | 38.92 | 15.21 | 56.00 | 0.59 | 0.09 | 1.09 | 5.62E-15 |
| CONGENITAL ANOMALIES OF EYE | 14.97 | -53.94 | 53.03 | 0.16 | -0.49 | 0.81 | 0.5921 |
| URO-GENITAL DEFECTS | -8.83 | -33.98 | 11.60 | -0.08 | -0.25 | 0.10 | 0.4244 |
| MUSCULO-SKELETAL DEFECTS | -15.65 | -35.11 | 1.00 | -0.13 | -0.25 | 0.00 | 0.0658 |
| CONGENITAL ANOMALIES OF INTEGUMENT | -18.22 | -42.69 | 2.05 | -0.14 | -0.29 | 0.00 | 1.39E-06 |
|  |  |  |  |  |  |  |  |
| NOT_CANNABIS_RELATED | -19.57 | -32.88 | -7.59 | -0.15 | -0.24 | -0.07 | 8.17E-04 |
| CANNABIS_RELATED | 32.79 | 26.07 | 38.91 | 0.45 | 0.32 | 0.59 | 5.39E-16 |

**Supplementary Table 5.: Regression Coefficients and Significance Levels for Defects against Tobacco, Alcohol and Cannabis Use**

**Ordered by Significance of Regression on Cannabis**

| **Defect** | **Tobacco Estimate** | **Tobacco P-value** | **Alcohol Estimate** | **Alcohol P-value** | **Cannabis Estimate** | **Cannabis P-Value** |
| --- | --- | --- | --- | --- | --- | --- |
|  |  |  |  |  |  |  |
| Small_Intestinal_Stenosis_Atresia | 0.0022 | 0.8560 | -0.0013 | 0.9970 | 0.0339 | 0.0059 |
| Turner_Syndrome | -0.0004 | 0.9600 | -0.1990 | 0.5120 | 0.0258 | 0.0102 |
| Tetralogy_Fallot | 0.0048 | 0.7460 | 0.1310 | 0.7740 | 0.0397 | 0.0107 |
| Hypospadias | -0.0231 | 0.0476 | -0.0904 | 0.8160 | -0.0319 | 0.0176 |
| Gastroschisis | 0.0041 | 0.8340 | 0.0557 | 0.9270 | 0.0487 | 0.0206 |
| Atrial_Septal_Defects | 0.0065 | 0.7710 | 0.1340 | 0.8460 | 0.0536 | 0.0272 |
| Congenital_hydrocephalus | 0.0085 | 0.5310 | 0.1640 | 0.7200 | 0.0319 | 0.0280 |
| Transposition_Great_Vessels | -0.0125 | 0.5300 | -0.4590 | 0.4630 | 0.0497 | 0.0338 |
| Diaphragmatic_Hernia | 0.0059 | 0.6780 | 0.0908 | 0.8370 | 0.0299 | 0.0596 |
| Anophthalmia | 0.0038 | 0.7090 | 0.1100 | 0.8080 | 0.0131 | 0.0629 |
| Patent_Ductus_Arteriosus | 0.0038 | 0.8570 | -0.3800 | 0.5570 | 0.0398 | 0.0928 |
| Cystic_Kidney_Disease | 0.0027 | 0.8450 | -0.2050 | 0.6540 | 0.0249 | 0.0994 |
| Exomphalos | -0.0077 | 0.6410 | -0.3150 | 0.5660 | 0.0315 | 0.1160 |
| CVS_Defects | 0.0067 | 0.6540 | 0.0567 | 0.9040 | 0.0261 | 0.1290 |
| CNS_Defects | 0.0161 | 0.2410 | 0.2270 | 0.6020 | 0.0243 | 0.1300 |
| CHROMOSOMAL_Defects | -0.0162 | 0.3390 | -0.5930 | 0.2560 | 0.0277 | 0.1590 |
| Syndactyly | -0.0113 | 0.4510 | -0.1110 | 0.8180 | -0.0236 | 0.1850 |
| Anotia_Microtia | 0.0029 | 0.7120 | -0.3020 | 0.2420 | 0.0131 | 0.1860 |
| Down_Syndrome | -0.0065 | 0.6950 | -0.9350 | 0.0534 | 0.0253 | 0.1880 |
| Trisomy_13 | -0.0068 | 0.2660 | -0.0236 | 0.9130 | 0.0101 | 0.2090 |
| Spina_Bifida | 0.0202 | 0.2220 | 0.3400 | 0.5150 | 0.0212 | 0.2820 |
| Trisomy_18 | -0.0174 | 0.3490 | -0.3290 | 0.6040 | 0.0229 | 0.2900 |
| Pyloric_Stenosis | 0.0083 | 0.6650 | -0.5270 | 0.4100 | -0.0228 | 0.3000 |
| Neural_tube_defects | 0.0234 | 0.1860 | 0.0759 | 0.8940 | 0.0212 | 0.3200 |
| Ventricular_Septal_Defect | -0.0148 | 0.4200 | -0.0323 | 0.9550 | 0.0206 | 0.3400 |
| Polydactyly | -0.0117 | 0.4730 | -0.2990 | 0.5880 | -0.0178 | 0.3470 |
| Limb_Reduction | -0.0123 | 0.3630 | 0.0985 | 0.8310 | -0.0144 | 0.3610 |
| Birth_Marks | -0.0886 | 0.0162 | -2.3600 | 0.0456 | -0.0425 | 0.3740 |
| Renal_Pelvis_Obstruction | -0.0017 | 0.9180 | 0.2450 | 0.6240 | 0.0158 | 0.4040 |
| SKIN_Defects | -0.0498 | 0.0721 | -1.3200 | 0.1320 | -0.0273 | 0.4280 |
| Ureteric_Anomalies | 0.0106 | 0.5140 | 0.2990 | 0.6020 | 0.0155 | 0.4510 |
| Talipes | -0.0205 | 0.3860 | -0.3140 | 0.6720 | -0.0197 | 0.4850 |
| Congenital cataract | 0.0073 | 0.3320 | -0.0694 | 0.7970 | 0.0061 | 0.5250 |
| GUT_Defects | -0.0062 | 0.3110 | -0.0829 | 0.6680 | -0.0047 | 0.5270 |
| MUSCULOSKELETAL_Defects | -0.0172 | 0.3160 | -0.3990 | 0.4570 | -0.0129 | 0.5320 |
| FACE_Defects | -0.0157 | 0.3200 | -0.4290 | 0.3830 | -0.0117 | 0.5380 |
| Hip_Dysplasia | -0.0367 | 0.1380 | -0.8920 | 0.2530 | -0.0185 | 0.5420 |
| Carbohydrate_Disorders | 0.0029 | 0.4470 | -0.0638 | 0.6680 | 0.0027 | 0.5530 |
| Coarctation_Aorta | -0.0018 | 0.9140 | 0.0001 | 1.0000 | 0.0109 | 0.5660 |
| Branchial_Remnants | -0.0049 | 0.6070 | 0.1230 | 0.6750 | 0.0066 | 0.5800 |
| Microcephaly | 0.0082 | 0.6520 | 0.5990 | 0.2800 | -0.0119 | 0.5950 |
| Amino_Acid_Disorders | -0.0017 | 0.8470 | -0.0652 | 0.8220 | -0.0045 | 0.6560 |
| TracheoEsophageal_Fistula | -0.0015 | 0.8490 | -0.5360 | 0.0348 | 0.0042 | 0.6600 |
| GIT_Defects | -0.0261 | 0.1540 | 0.0334 | 0.9550 | -0.0092 | 0.6830 |
| Undescended_Testis | -0.0054 | 0.5650 | -0.2890 | 0.3180 | 0.0045 | 0.6860 |
| Anal_Stenosis_Atresia | 0.0124 | 0.1880 | -0.6760 | 0.0225 | -0.0042 | 0.7090 |
| EYE_Defects | 0.0041 | 0.8320 | -0.5100 | 0.3840 | 0.0084 | 0.7110 |
| Anencephalus | 0.0123 | 0.3690 | -0.0286 | 0.9470 | 0.0060 | 0.7140 |
| Renal_Agenesis | -0.0063 | 0.6500 | 0.2160 | 0.6130 | -0.0060 | 0.7140 |
| Microphthalmia | 0.0010 | 0.8600 | -0.4150 | 0.0317 | -0.0022 | 0.7400 |
| Cleft_Palate&Lip | 0.0034 | 0.7440 | -0.2340 | 0.4640 | -0.0035 | 0.7740 |
| RESPIRATORY Defects | -0.0202 | 0.0907 | -0.6590 | 0.0736 | -0.0043 | 0.7750 |
| Craniosynostosis | 0.0128 | 0.3150 | -0.0110 | 0.9780 | -0.0043 | 0.7810 |
| Encephalocoele | -0.0008 | 0.8240 | 0.1220 | 0.3180 | 0.0014 | 0.7830 |
| Choanal_Atresia | -0.0093 | 0.2370 | -0.4030 | 0.1130 | 0.0014 | 0.8920 |

**Supplementary Table 6.: Regression Coefficients and Significance Levels for Defects against Tobacco, Alcohol and Cannabis Use**

**Ordered by Significance of Regression on Tobacco**

| **Defect** | **Tobacco Estimate** | **Tobacco P-value** | **Alcohol Estimate** | **Alcohol P-value** | **Cannabis Estimate** | **Cannabis P-Value** |
| --- | --- | --- | --- | --- | --- | --- |
|  |  |  |  |  |  |  |
| Birth_Marks | -0.0886 | 0.0162 | -2.3600 | 0.0456 | -0.0425 | 0.3740 |
| Hypospadias | -0.0231 | 0.0476 | -0.0904 | 0.8160 | -0.0319 | 0.0176 |
| SKIN_Defects | -0.0498 | 0.0721 | -1.3200 | 0.1320 | -0.0273 | 0.4280 |
| RESPIRATORY_Defects | -0.0202 | 0.0907 | -0.6590 | 0.0736 | -0.0043 | 0.7750 |
| Hip_Dysplasia | -0.0367 | 0.1380 | -0.8920 | 0.2530 | -0.0185 | 0.5420 |
| GIT_Defects | -0.0261 | 0.1540 | 0.0334 | 0.9550 | -0.0092 | 0.6830 |
| Neural_tube_defects | 0.0234 | 0.1860 | 0.0759 | 0.8940 | 0.0212 | 0.3200 |
| Anal_Stenosis_Atresia | 0.0124 | 0.1880 | -0.6760 | 0.0225 | -0.0042 | 0.7090 |
| Spina_Bifida | 0.0202 | 0.2220 | 0.3400 | 0.5150 | 0.0212 | 0.2820 |
| Choanal_Atresia | -0.0093 | 0.2370 | -0.4030 | 0.1130 | 0.0014 | 0.8920 |
| CNS_Defects | 0.0161 | 0.2410 | 0.2270 | 0.6020 | 0.0243 | 0.1300 |
| Trisomy_13 | -0.0068 | 0.2660 | -0.0236 | 0.9130 | 0.0101 | 0.2090 |
| GUT_Defects | -0.0062 | 0.3110 | -0.0829 | 0.6680 | -0.0047 | 0.5270 |
| Craniosynostosis | 0.0128 | 0.3150 | -0.0110 | 0.9780 | -0.0043 | 0.7810 |
| MUSCULOSKELETAL_Defects | -0.0172 | 0.3160 | -0.3990 | 0.4570 | -0.0129 | 0.5320 |
| FACE_Defects | -0.0157 | 0.3200 | -0.4290 | 0.3830 | -0.0117 | 0.5380 |
| Congenital cataract | 0.0073 | 0.3320 | -0.0694 | 0.7970 | 0.0061 | 0.5250 |
| CHROMOSOMAL_Defects | -0.0162 | 0.3390 | -0.5930 | 0.2560 | 0.0277 | 0.1590 |
| Trisomy_18 | -0.0174 | 0.3490 | -0.3290 | 0.6040 | 0.0229 | 0.2900 |
| Limb_Reduction | -0.0123 | 0.3630 | 0.0985 | 0.8310 | -0.0144 | 0.3610 |
| Anencephalus | 0.0123 | 0.3690 | -0.0286 | 0.9470 | 0.0060 | 0.7140 |
| Talipes | -0.0205 | 0.3860 | -0.3140 | 0.6720 | -0.0197 | 0.4850 |
| Ventricular_Septal_Defect | -0.0148 | 0.4200 | -0.0323 | 0.9550 | 0.0206 | 0.3400 |
| Carbohydrate_Disorders | 0.0029 | 0.4470 | -0.0638 | 0.6680 | 0.0027 | 0.5530 |
| Syndactyly | -0.0113 | 0.4510 | -0.1110 | 0.8180 | -0.0236 | 0.1850 |
| Polydactyly | -0.0117 | 0.4730 | -0.2990 | 0.5880 | -0.0178 | 0.3470 |
| Ureteric_Anomalies | 0.0106 | 0.5140 | 0.2990 | 0.6020 | 0.0155 | 0.4510 |
| Transposition_Great_Vessels | -0.0125 | 0.5300 | -0.4590 | 0.4630 | 0.0497 | 0.0338 |
| Congenital_hydrocephalus | 0.0085 | 0.5310 | 0.1640 | 0.7200 | 0.0319 | 0.0280 |
| Undescended_Testis | -0.0054 | 0.5650 | -0.2890 | 0.3180 | 0.0045 | 0.6860 |
| Branchial_Remnants | -0.0049 | 0.6070 | 0.1230 | 0.6750 | 0.0066 | 0.5800 |
| Exomphalos | -0.0077 | 0.6410 | -0.3150 | 0.5660 | 0.0315 | 0.1160 |
| Renal_Agenesis | -0.0063 | 0.6500 | 0.2160 | 0.6130 | -0.0060 | 0.7140 |
| Microcephaly | 0.0082 | 0.6520 | 0.5990 | 0.2800 | -0.0119 | 0.5950 |
| CVS_Defects | 0.0067 | 0.6540 | 0.0567 | 0.9040 | 0.0261 | 0.1290 |
| Pyloric_Stenosis | 0.0083 | 0.6650 | -0.5270 | 0.4100 | -0.0228 | 0.3000 |
| Diaphragmatic_Hernia | 0.0059 | 0.6780 | 0.0908 | 0.8370 | 0.0299 | 0.0596 |
| Down_Syndrome | -0.0065 | 0.6950 | -0.9350 | 0.0534 | 0.0253 | 0.1880 |
| Anophthalmia | 0.0038 | 0.7090 | 0.1100 | 0.8080 | 0.0131 | 0.0629 |
| Anotia_Microtia | 0.0029 | 0.7120 | -0.3020 | 0.2420 | 0.0131 | 0.1860 |
| Cleft_Palate&Lip | 0.0034 | 0.7440 | -0.2340 | 0.4640 | -0.0035 | 0.7740 |
| Tetralogy_Fallot | 0.0048 | 0.7460 | 0.1310 | 0.7740 | 0.0397 | 0.0107 |
| Atrial_Septal_Defects | 0.0065 | 0.7710 | 0.1340 | 0.8460 | 0.0536 | 0.0272 |
| Encephalocoele | -0.0008 | 0.8240 | 0.1220 | 0.3180 | 0.0014 | 0.7830 |
| EYE_Defects | 0.0041 | 0.8320 | -0.5100 | 0.3840 | 0.0084 | 0.7110 |
| Gastroschisis | 0.0041 | 0.8340 | 0.0557 | 0.9270 | 0.0487 | 0.0206 |
| Cystic_Kidney_Disease | 0.0027 | 0.8450 | -0.2050 | 0.6540 | 0.0249 | 0.0994 |
| Amino_Acid_Disorders | -0.0017 | 0.8470 | -0.0652 | 0.8220 | -0.0045 | 0.6560 |
| TracheoEsophageal_Fistula | -0.0015 | 0.8490 | -0.5360 | 0.0348 | 0.0042 | 0.6600 |
| Small_Intestinal_Stenosis_Atresia | 0.0022 | 0.8560 | -0.0013 | 0.9970 | 0.0339 | 0.0059 |
| Patent_Ductus_Arteriosus | 0.0038 | 0.8570 | -0.3800 | 0.5570 | 0.0398 | 0.0928 |
| Microphthalmia | 0.0010 | 0.8600 | -0.4150 | 0.0317 | -0.0022 | 0.7400 |
| Coarctation_Aorta | -0.0018 | 0.9140 | 0.0001 | 1.0000 | 0.0109 | 0.5660 |
| Renal_Pelvis_Obstruction | -0.0017 | 0.9180 | 0.2450 | 0.6240 | 0.0158 | 0.4040 |
| Turner_Syndrome | -0.0004 | 0.9600 | -0.1990 | 0.5120 | 0.0258 | 0.0102 |

**Supplementary Table 7.: Regression Coefficients and Significance Levels for Defects against Tobacco, Alcohol and Cannabis Use**

**Ordered by Significance of Regression on Alcohol**

| **Defect** | **Tobacco Estimate** | **Tobacco P-value** | **Alcohol Estimate** | **Alcohol P-value** | **Cannabis Estimate** | **Cannabis P-Value** |
| --- | --- | --- | --- | --- | --- | --- |
|  |  |  |  |  |  |  |
| Anal_Stenosis_Atresia | 0.0124 | 0.1880 | -0.6760 | 0.0225 | -0.0042 | 0.7090 |
| Microphthalmia | 0.0010 | 0.8600 | -0.4150 | 0.0317 | -0.0022 | 0.7400 |
| TracheoEsophageal_Fistula | -0.0015 | 0.8490 | -0.5360 | 0.0348 | 0.0042 | 0.6600 |
| Birth_Marks | -0.0886 | 0.0162 | -2.3600 | 0.0456 | -0.0425 | 0.3740 |
| Down_Syndrome | -0.0065 | 0.6950 | -0.9350 | 0.0534 | 0.0253 | 0.1880 |
| RESPIRATORY_Defects | -0.0202 | 0.0907 | -0.6590 | 0.0736 | -0.0043 | 0.7750 |
| Choanal_Atresia | -0.0093 | 0.2370 | -0.4030 | 0.1130 | 0.0014 | 0.8920 |
| SKIN_Defects | -0.0498 | 0.0721 | -1.3200 | 0.1320 | -0.0273 | 0.4280 |
| Anotia_Microtia | 0.0029 | 0.7120 | -0.3020 | 0.2420 | 0.0131 | 0.1860 |
| Hip_Dysplasia | -0.0367 | 0.1380 | -0.8920 | 0.2530 | -0.0185 | 0.5420 |
| CHROMOSOMAL_Defects | -0.0162 | 0.3390 | -0.5930 | 0.2560 | 0.0277 | 0.1590 |
| Microcephaly | 0.0082 | 0.6520 | 0.5990 | 0.2800 | -0.0119 | 0.5950 |
| Undescended_Testis | -0.0054 | 0.5650 | -0.2890 | 0.3180 | 0.0045 | 0.6860 |
| Encephalocoele | -0.0008 | 0.8240 | 0.1220 | 0.3180 | 0.0014 | 0.7830 |
| FACE_Defects | -0.0157 | 0.3200 | -0.4290 | 0.3830 | -0.0117 | 0.5380 |
| EYE_Defects | 0.0041 | 0.8320 | -0.5100 | 0.3840 | 0.0084 | 0.7110 |
| Pyloric_Stenosis | 0.0083 | 0.6650 | -0.5270 | 0.4100 | -0.0228 | 0.3000 |
| MUSCULOSKELETAL_Defects | -0.0172 | 0.3160 | -0.3990 | 0.4570 | -0.0129 | 0.5320 |
| Transposition_Great_Vessels | -0.0125 | 0.5300 | -0.4590 | 0.4630 | 0.0497 | 0.0338 |
| Cleft_Palate&Lip | 0.0034 | 0.7440 | -0.2340 | 0.4640 | -0.0035 | 0.7740 |
| Turner_Syndrome | -0.0004 | 0.9600 | -0.1990 | 0.5120 | 0.0258 | 0.0102 |
| Spina_Bifida | 0.0202 | 0.2220 | 0.3400 | 0.5150 | 0.0212 | 0.2820 |
| Patent_Ductus_Arteriosus | 0.0038 | 0.8570 | -0.3800 | 0.5570 | 0.0398 | 0.0928 |
| Exomphalos | -0.0077 | 0.6410 | -0.3150 | 0.5660 | 0.0315 | 0.1160 |
| Polydactyly | -0.0117 | 0.4730 | -0.2990 | 0.5880 | -0.0178 | 0.3470 |
| CNS_Defects | 0.0161 | 0.2410 | 0.2270 | 0.6020 | 0.0243 | 0.1300 |
| Ureteric_Anomalies | 0.0106 | 0.5140 | 0.2990 | 0.6020 | 0.0155 | 0.4510 |
| Trisomy_18 | -0.0174 | 0.3490 | -0.3290 | 0.6040 | 0.0229 | 0.2900 |
| Renal_Agenesis | -0.0063 | 0.6500 | 0.2160 | 0.6130 | -0.0060 | 0.7140 |
| Renal_Pelvis_Obstruction | -0.0017 | 0.9180 | 0.2450 | 0.6240 | 0.0158 | 0.4040 |
| Cystic_Kidney_Disease | 0.0027 | 0.8450 | -0.2050 | 0.6540 | 0.0249 | 0.0994 |
| GUT_Defects | -0.0062 | 0.3110 | -0.0829 | 0.6680 | -0.0047 | 0.5270 |
| Carbohydrate_Disorders | 0.0029 | 0.4470 | -0.0638 | 0.6680 | 0.0027 | 0.5530 |
| Talipes | -0.0205 | 0.3860 | -0.3140 | 0.6720 | -0.0197 | 0.4850 |
| Branchial_Remnants | -0.0049 | 0.6070 | 0.1230 | 0.6750 | 0.0066 | 0.5800 |
| Congenital_hydrocephalus | 0.0085 | 0.5310 | 0.1640 | 0.7200 | 0.0319 | 0.0280 |
| Tetralogy_Fallot | 0.0048 | 0.7460 | 0.1310 | 0.7740 | 0.0397 | 0.0107 |
| Congenital cataract | 0.0073 | 0.3320 | -0.0694 | 0.7970 | 0.0061 | 0.5250 |
| Anophthalmia | 0.0038 | 0.7090 | 0.1100 | 0.8080 | 0.0131 | 0.0629 |
| Hypospadias | -0.0231 | 0.0476 | -0.0904 | 0.8160 | -0.0319 | 0.0176 |
| Syndactyly | -0.0113 | 0.4510 | -0.1110 | 0.8180 | -0.0236 | 0.1850 |
| Amino_Acid_Disorders | -0.0017 | 0.8470 | -0.0652 | 0.8220 | -0.0045 | 0.6560 |
| Limb_Reduction | -0.0123 | 0.3630 | 0.0985 | 0.8310 | -0.0144 | 0.3610 |
| Diaphragmatic_Hernia | 0.0059 | 0.6780 | 0.0908 | 0.8370 | 0.0299 | 0.0596 |
| Atrial_Septal_Defects | 0.0065 | 0.7710 | 0.1340 | 0.8460 | 0.0536 | 0.0272 |
| Neural_tube_defects | 0.0234 | 0.1860 | 0.0759 | 0.8940 | 0.0212 | 0.3200 |
| CVS_Defects | 0.0067 | 0.6540 | 0.0567 | 0.9040 | 0.0261 | 0.1290 |
| Trisomy_13 | -0.0068 | 0.2660 | -0.0236 | 0.9130 | 0.0101 | 0.2090 |
| Gastroschisis | 0.0041 | 0.8340 | 0.0557 | 0.9270 | 0.0487 | 0.0206 |
| Anencephalus | 0.0123 | 0.3690 | -0.0286 | 0.9470 | 0.0060 | 0.7140 |
| GIT_Defects | -0.0261 | 0.1540 | 0.0334 | 0.9550 | -0.0092 | 0.6830 |
| Ventricular_Septal_Defect | -0.0148 | 0.4200 | -0.0323 | 0.9550 | 0.0206 | 0.3400 |
| Craniosynostosis | 0.0128 | 0.3150 | -0.0110 | 0.9780 | -0.0043 | 0.7810 |
| Small_Intestinal_Stenosis_Atresia | 0.0022 | 0.8560 | -0.0013 | 0.9970 | 0.0339 | 0.0059 |
| Coarctation_Aorta | -0.0018 | 0.9140 | 0.0001 | 1.0000 | 0.0109 | 0.5660 |

**Supplementary Table 8.: Expanded Outcomes from Additive Linear Modelling of 3 Drug Exposure – P<0.3**

| **Defect** | **Term** | **Estimate** | **Std.Error** | **t** | **P** | **P_Holm** | **P_Bonf** | **P_BY** | **P_FDR** | **P_Hommel** |
| --- | --- | --- | --- | --- | --- | --- | --- | --- | --- | --- |
|  |  |  |  |  |  |  |  |  |  |  |
| Anal_Stenosis_Atresia | Binge_Alcohol | -0.8160 | 0.2180 | -3.7500 | 0.0032 | 0.1510 | 0.1510 | 0.4770 | 0.1070 | 0.1480 |
| Turner_Syndrome | Cannabis | 0.0305 | 0.0078 | 3.9000 | 0.0046 | 0.2100 | 0.2150 | 0.4770 | 0.1070 | 0.1990 |
| Small_Intestinal_Stenosis_Atresia | Cannabis | 0.0372 | 0.0116 | 3.2100 | 0.0075 | 0.3380 | 0.3530 | 0.5230 | 0.1180 | 0.2710 |
| Anal_Stenosis_Atresia | Tobacco | 0.0204 | 0.0067 | 3.0400 | 0.0112 | 0.4920 | 0.5260 | 0.5830 | 0.1310 | 0.2950 |
| Transposition_Great_Vessels | Cannabis | 0.0595 | 0.0205 | 2.9000 | 0.0157 | 0.6760 | 0.7390 | 0.5940 | 0.1340 | 0.2950 |
| Tetralogy_Fallot | Cannabis | 0.0422 | 0.0153 | 2.7700 | 0.0171 | 0.7180 | 0.8030 | 0.5940 | 0.1340 | 0.2950 |
| Gastroschisis | Cannabis | 0.0529 | 0.0211 | 2.5100 | 0.0273 | 1 | 1 | 0.7770 | 0.1750 | 0.2950 |
| Down_Syndrome | Binge_Alcohol | -1.0400 | 0.4340 | -2.3900 | 0.0343 | 1 | 1 | 0.7770 | 0.1750 | 0.2950 |
| Atrial_Septal_Defects | Cannabis | 0.0571 | 0.0247 | 2.3200 | 0.0391 | 1 | 1 | 0.7770 | 0.1750 | 0.2950 |
| TracheoEsophageal_Fistula | Binge_Alcohol | -0.5670 | 0.2470 | -2.3000 | 0.0444 | 1 | 1 | 0.7770 | 0.1750 | 0.2950 |
| CHROMOSOMAL_Defects | Cannabis | 0.0396 | 0.0183 | 2.1700 | 0.0512 | 1 | 1 | 0.7770 | 0.1750 | 0.2950 |
| Hypospadias | Cannabis | -0.0264 | 0.0122 | -2.1600 | 0.0514 | 1 | 1 | 0.7770 | 0.1750 | 0.2950 |
| Congenital_hydrocephalus | Cannabis | 0.0321 | 0.0149 | 2.1500 | 0.0547 | 1 | 1 | 0.7770 | 0.1750 | 0.2950 |
| Microphthalmia | Binge_Alcohol | -0.4140 | 0.1860 | -2.2300 | 0.0611 | 1 | 1 | 0.7770 | 0.1750 | 0.2950 |
| Exomphalos | Cannabis | 0.0417 | 0.0196 | 2.1200 | 0.0628 | 1 | 1 | 0.7770 | 0.1750 | 0.2950 |
| Down_Syndrome | Cannabis | 0.0342 | 0.0167 | 2.0500 | 0.0629 | 1 | 1 | 0.7770 | 0.1750 | 0.2950 |
| Birth_Marks | Tobacco | -0.0711 | 0.0347 | -2.0500 | 0.0633 | 1 | 1 | 0.7770 | 0.1750 | 0.2950 |
| Diaphragmatic_Hernia | Cannabis | 0.0309 | 0.0167 | 1.8500 | 0.0884 | 1 | 1 | 0.9410 | 0.2120 | 0.2950 |
| Patent_Ductus_Arteriosus | Cannabis | 0.0445 | 0.0243 | 1.8300 | 0.0926 | 1 | 1 | 0.9410 | 0.2120 | 0.2950 |
| Anophthalmia | Cannabis | 0.0187 | 0.0064 | 2.9200 | 0.1000 | 1 | 1 | 0.9410 | 0.2120 | 0.2950 |
| Cystic_Kidney_Disease | Cannabis | 0.0280 | 0.0157 | 1.7800 | 0.1030 | 1 | 1 | 0.9410 | 0.2120 | 0.2950 |
| Anotia_Microtia | Binge_Alcohol | -0.4360 | 0.2370 | -1.8400 | 0.1030 | 1 | 1 | 0.9410 | 0.2120 | 0.2950 |
| Trisomy_13 | Cannabis | 0.0136 | 0.0077 | 1.7800 | 0.1130 | 1 | 1 | 0.9410 | 0.2120 | 0.2950 |
| Anotia_Microtia | Cannabis | 0.0161 | 0.0091 | 1.7700 | 0.1150 | 1 | 1 | 0.9410 | 0.2120 | 0.2950 |
| Turner_Syndrome | Binge_Alcohol | -0.3580 | 0.2030 | -1.7600 | 0.1160 | 1 | 1 | 0.9410 | 0.2120 | 0.2950 |
| Hypospadias | Tobacco | -0.0179 | 0.0106 | -1.6900 | 0.1170 | 1 | 1 | 0.9410 | 0.2120 | 0.2950 |
| Birth_Marks | Binge_Alcohol | -1.6700 | 1.0400 | -1.6100 | 0.1330 | 1 | 1 | 1 | 0.2320 | 0.2950 |
| Trisomy_18 | Cannabis | 0.0341 | 0.0218 | 1.5600 | 0.1460 | 1 | 1 | 1 | 0.2370 | 0.2950 |
| Trisomy_13 | Tobacco | -0.0093 | 0.0058 | -1.6100 | 0.1460 | 1 | 1 | 1 | 0.2370 | 0.2950 |
| Choanal_Atresia | Binge_Alcohol | -0.3960 | 0.2580 | -1.5400 | 0.1590 | 1 | 1 | 1 | 0.2490 | 0.2950 |
| RESPIRATORY_Defects | Binge_Alcohol | -0.5280 | 0.3610 | -1.4600 | 0.1690 | 1 | 1 | 1 | 0.2540 | 0.2950 |
| GIT_Defects | Tobacco | -0.0291 | 0.0203 | -1.4400 | 0.1760 | 1 | 1 | 1 | 0.2540 | 0.2950 |
| CVS_Defects | Cannabis | 0.0264 | 0.0185 | 1.4300 | 0.1790 | 1 | 1 | 1 | 0.2540 | 0.2950 |
| RESPIRATORY_Defects | Tobacco | -0.0165 | 0.0121 | -1.3700 | 0.1970 | 1 | 1 | 1 | 0.2690 | 0.2950 |
| SKIN_Defects | Tobacco | -0.0390 | 0.0288 | -1.3500 | 0.2010 | 1 | 1 | 1 | 0.2690 | 0.2950 |
| CHROMOSOMAL_Defects | Tobacco | -0.0213 | 0.0159 | -1.3400 | 0.2060 | 1 | 1 | 1 | 0.2690 | 0.2950 |
| Trisomy_18 | Tobacco | -0.0246 | 0.0190 | -1.3000 | 0.2220 | 1 | 1 | 1 | 0.2750 | 0.2950 |
| Ventricular_Septal_Defect | Cannabis | 0.0291 | 0.0226 | 1.2900 | 0.2230 | 1 | 1 | 1 | 0.2750 | 0.2950 |
| CHROMOSOMAL_Defects | Binge_Alcohol | -0.5690 | 0.4750 | -1.2000 | 0.2540 | 1 | 1 | 1 | 0.2940 | 0.2950 |
| CNS_Defects | Cannabis | 0.0201 | 0.0169 | 1.1900 | 0.2560 | 1 | 1 | 1 | 0.2940 | 0.2950 |
| Ventricular_Septal_Defect | Tobacco | -0.0232 | 0.0197 | -1.1800 | 0.2620 | 1 | 1 | 1 | 0.2940 | 0.2950 |
| Craniosynostosis | Tobacco | 0.0166 | 0.0142 | 1.1700 | 0.2650 | 1 | 1 | 1 | 0.2940 | 0.2950 |
| Pyloric_Stenosis | Cannabis | -0.0268 | 0.0230 | -1.1600 | 0.2690 | 1 | 1 | 1 | 0.2940 | 0.2950 |
| Transposition_Great_Vessels | Tobacco | -0.0192 | 0.0166 | -1.1500 | 0.2760 | 1 | 1 | 1 | 0.2940 | 0.2950 |
| Syndactyly | Cannabis | -0.0214 | 0.0192 | -1.1200 | 0.2880 | 1 | 1 | 1 | 0.2940 | 0.2950 |
| Hip_Dysplasia | Tobacco | -0.0300 | 0.0270 | -1.1100 | 0.2880 | 1 | 1 | 1 | 0.2940 | 0.2950 |
| Neural_tube_defects | Tobacco | 0.0215 | 0.0196 | 1.1000 | 0.2950 | 1 | 1 | 1 | 0.2950 | 0.2950 |

**Supplementary Table 9.: Linear Interactive Final Models**

| **Parameter** | | | **Model** | | | |
| --- | --- | --- | --- | --- | --- | --- |
| **Parameter** | **Estimate (C.I.)** | **P-Value** | **Adj R-Squared** | **F** | **Degrees Freedom, 1,2** | **Model P** |
|  |  |  |  |  |  |  |
| ***lm(Rate ~ Cigarettes * Binge_Alcohol * Cannabis)*** | | | | | | |
|  |  |  |  |  |  |  |
| ***Anal Stenosis / Atresia*** |  |  |  |  |  |  |
| Binge_Alcohol: Cannabis | -0.21 (-0.33, -0.1) | 0.0042 | 0.4564 | 4.918 | 3,11 | 0.0209 |
| Cannabis | 0.62 (0.25, 0.99) | 0.0075 |  |  |  |  |
| Tobacco: Binge_Alcohol: Cannabis | 0 (0, 0) | 0.0141 |  |  |  |  |
|  |  |  |  |  |  |  |
| ***Anotia / Microtia*** |  |  |  |  |  |  |
| Cannabis | 0.21 (0.07, 0.35) | 0.0198 | 0.422 | 3.008 | 4,7 | 0.0970 |
| Binge_Alcohol: Cannabis | -0.08 (-0.13, -0.03) | 0.0207 |  |  |  |  |
|  |  |  |  |  |  |  |
| ***Congenital Hydrocephalus*** |  |  |  |  |  |  |
| Cannabis | 0.05 (0.01, 0.09) | 0.0268 | 0.2719 | 6.227 | 1,13 | 0.0268 |
|  |  |  |  |  |  |  |
| ***Exomphalos*** |  |  |  |  |  |  |
| Binge_Alcohol: Cannabis | 0.05 (0, 0.09) | 0.0765 | 0.1536 | 2.089 | 2,10 | 0.1746 |
| Tobacco: Binge_Alcohol: Cannabis | 0 (0, 0) | 0.0783 |  |  |  |  |
|  |  |  |  |  |  |  |
| ***Microphthalmia*** |  |  |  |  |  |  |
| Tobacco: Binge_Alcohol: Cannabis | 0 (0, 0) | 0.0302 | 0.3375 | 3.547 | 2,8 | 0.0789 |
| Tobacco: Cannabis | 0.01 (0, 0.01) | 0.0321 |  |  |  |  |
|  |  |  |  |  |  |  |
| ***Transposition_Great_Vessels*** |  |  |  |  |  |  |
| Binge_Alcohol: Cannabis | 0.03 (0.01, 0.05) | 0.0336 | 0.2761 | 3.479 | 2,11 | 0.0675 |
|  |  |  |  |  |  |  |
| ***Turner_Syndrome*** |  |  |  |  |  |  |
| Binge_Alcohol: Cannabis | 0.02 (0.02, 0.03) | 0.0008 | 0.6937 | 13.46 | 2,9 | 0.0020 |
| Binge_Alcohol | -1.46 (-2.35, -0.57) | 0.0106 |  |  |  |  |
|  |  |  |  |  |  |  |
| ***Trisomy_13*** |  |  |  |  |  |  |
| Tobacco: Binge_Alcohol |  |  | 0.2917 | 3.265 | 2,9 | 0.0859 |
| Tobacco: Binge_Alcohol: Cannabis | 0 (0, 0) | 0.0537 |  |  |  |  |
