# Supplementary material for: Broad Spectrum epidemiological contribution of cannabis and other substances to the teratological profile of northern New South Wales: geospatial and causal inference analysis
Source: BMC Pharmacol Toxicol. 2020 Nov 12;21:75. doi: 10.1186/s40360-020-00450-1 (PMC7659114; doi:10.1186/s40360-020-00450-1)
Supplement: Supplementary file 2 — Additional file 2 Supplementary Fig. 1.: Choropleth maps of congenital anomaly class rates across QLD and NNSW for Congenital anopmalies A-C. High rates are shown in yellow and low rates in dark blue. Maps were drawn using R package “sf” [15]. Supplementary Fig. 2.: Choropleth maps of congenital anomaly class rates across QLD and NNSW for Congenital anopmalies C-P. High rates are shown in yellow and low rates in dark blue. Maps were drawn using R package “sf” [15]. Supplementary Fig. 3.: Choropleth maps of congenital anomaly class rates across QLD and NNSW for Congenital anopmalies R-Z. High rates are shown in yellow and low rates in dark blue. Maps were drawn using R package “sf” [15]. [file 40360_2020_450_MOESM2_ESM.zip › SFig. 2 - CAs C-PR2.pdf]

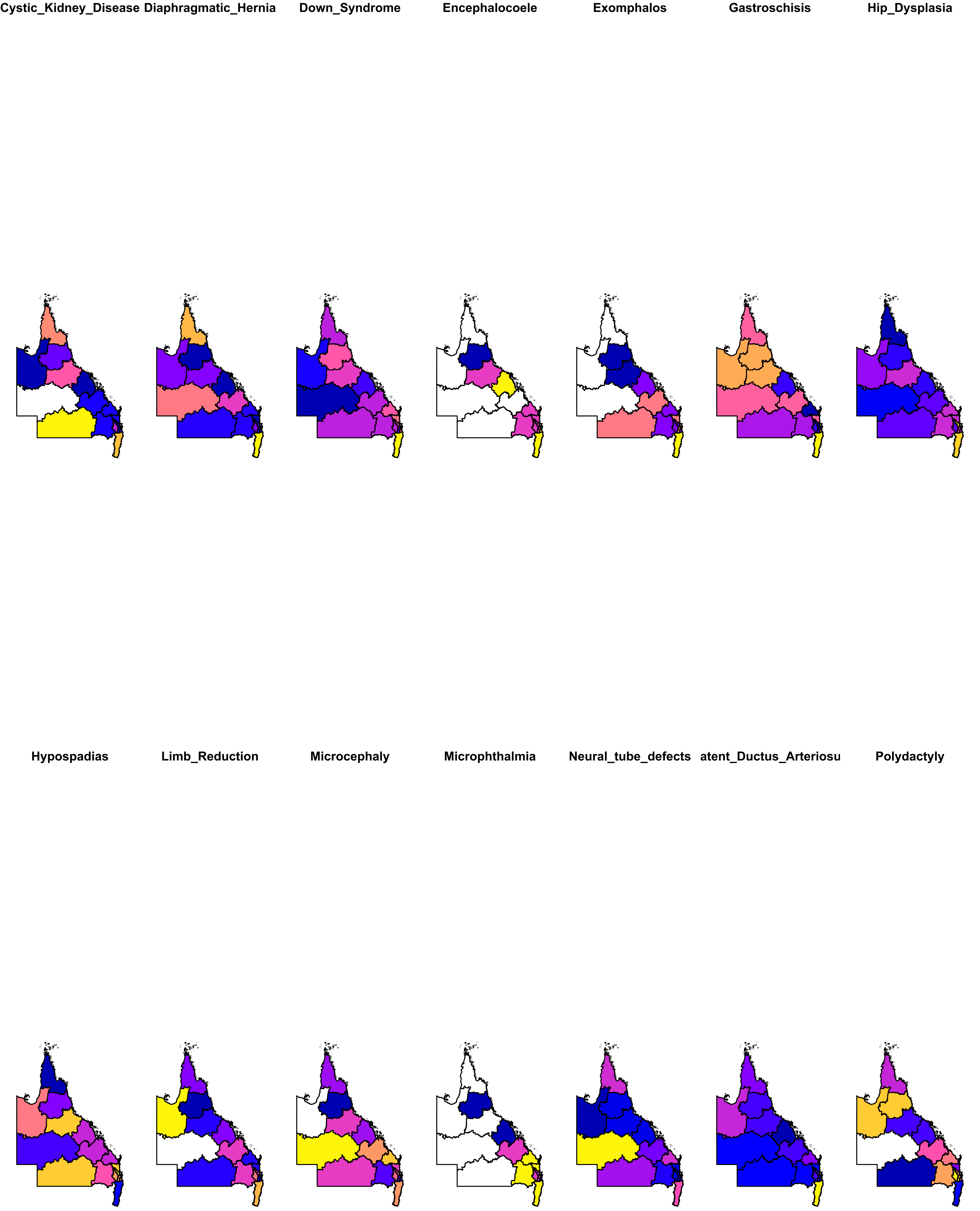

Cystic_Kidney_Disease
Diaphragmatic_Hernia
Down_Syndrome
Encephalocoele
Exomphalos
Gastroschisis
Hip_Dysplasia
Hypospadias
Limb_Reduction
Microcephaly
Microphthalmia
Neural_tube_defects
atent_Ductus_Arteriosu
Polydactyly
